# Probing the Origin of Challenge of Realizing Metallaphosphabenzenes: Unfavorable 1,2-Migration in Metallapyridines Becomes Feasible in Metallaphosphabenzenes

**DOI:** 10.1038/srep28543

**Published:** 2016-06-24

**Authors:** Jingjing Wu, Yulei Hao, Jun Zhu

**Affiliations:** 1 State Key Laboratory of Physical Chemistry of Solid Surfaces, Collaborative Innovation Center of Chemistry for Energy Materials (iChEM) and Fujian Provincial Key Laboratory of Theoretical and Computational Chemistry, College of Chemistry and Chemical Engineering, Xiamen University, Xiamen, Fujian 361005, China

## Abstract

Metallabenzenes have attracted considerable interest of both theoretical and experimental chemists. However, metallaphosphabenzene has never been synthesized. Thus, understanding the origin of the challenge of synthesizing metallaphosphabenzene is particularly urgent for experimentalists. Now density functional theory (DFT) calculations have been carried out to examine this issue. Our results reveal that the 1,2-migration in metallapyridines is unfavorable whereas such a 1,2-migration in metallaphosphabenzenes is feasible, which can be rationalized by the reluctance of phosphorus to participate in π bonding. In addition, π-donor ligands and the *5d* transition metals can stabilize metallaphosphabenzenes. Compared with hydride and methyl migration, the chloride migration has a relatively lower activation barrier due to the polarization of the M=P bond. CO ligand could further decrease the reaction barrier of the migration due to the reduction of the interaction between the metal centre and the phosphorus atom. All of these findings could help synthetic chemists to realize the first metallaphosphabenzene.

The chemistry of transition-metal-containing aromatics has attracted continuously increasing attention^1^,^2^,^3^,^4^,^5^,^6^ since metallabenzene was first predicted by Hoffmann^7^ and isolated by Roper^8^. In contrast, the heteroatom-containing metallabenzenes such as metallapyrylium^9^,^10^, metallathiabenzene^11^,^12^, metallapyridine^13^,^14^, metallapyrrole^15^,^16^, and metallathiophene^17^,^18^ are relatively less developed. To the best of our knowledge, metallaphosphabenzene has not been synthesized so far, although its structure and property were investigated theoretically^19^,^20^. The difficulties in the synthesis and isolation of metallaphosphabenzene could be due to the reluctance of phosphorus to participate in multiple bonds^21^, similar to our previous finding^22^ that 1,2-migration in metallasilabenzenes becomes favorable due to the reluctance of silicon to participate in π bonding. Thus, 1,2-migration in metallaphosphabenzenes may also occur (Fig. 1A). Another reason for the difficulties in the synthesis of metallaphosphabenzene could be facile isomerization of metallaphosphabenzene **II** to the corresponding η^5^-phosphacyclopentadiene (PCp) metal complex **I**, similar to that of metallabenzenes^23^^,^^24^. Indeed, our previous study^20^ shows that substituents have a significant effect on the thermodynamics and kinetics of the rearrangement reactions. Thus, an interconversion between osmaphosphabenzenes and the corresponding η^5^-PCp complexes can be achieved theoretically by simply tuning the substituents on the metallacycles. In comparison, the isomerization from metallaphosphabenzene **II** to nonaromatic analogue **III** has never been reported. Our ongoing interest in aromaticity^25^,^26^,^27^,^28^,^29^,^30^,^31^,^32^ and reaction mechanisms^33^,^34^,^35^,^36^,^37^ has led us to test the hypothetical isomerization of metallaphosphabenzene **II** to the nonaromatic analogue **III** as described in Fig 1A. Here we carry out thorough density function theory (DFT) calculations on this issue. How the ligands, metal centers affect the reaction mechanisms will be investigated in detail.

## Results

### Thermodynamic aspect of chloride migration

**Figure 1:**
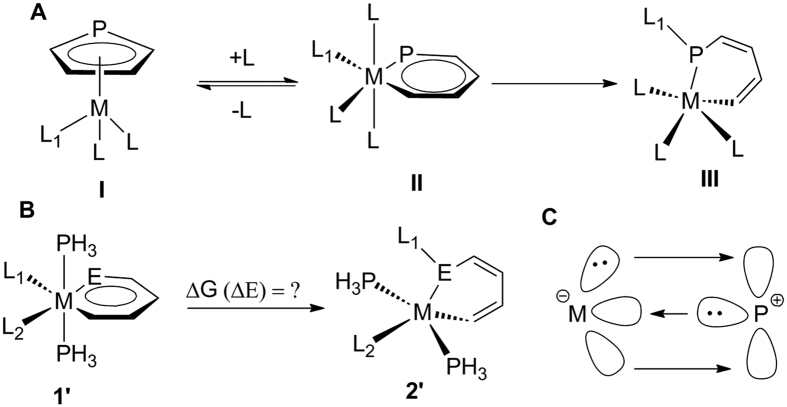
(**A**) Isomerizations from metallaphosphabenzenes (**II**) to η^5^-PCp metal complex (**I**) or non-aromatic complex (**III**). (**B**) 1,2-Migration of metallapyridines (E=N) and metallaphosphabenzenes (E=P). (**C**) Schematic diagram of the M=P bonding in phosphinidene complex.

**Figure 2:**
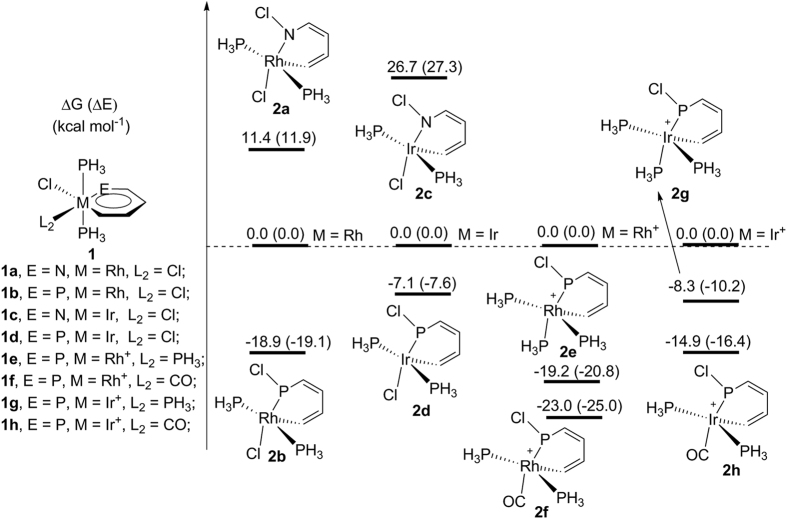
Thermodynamic stabilities of metallaphosphabenzene (**1a**–**1h**) compared with its nonaromatic analogues (**2a**–**2h**) formed by chloride migration.

In this work, we focus on *ο*-metallaphosphabenzenes (Fig. 2, **1a**–**h**) as a phosphorus atom at the ortho position on metallaphosphabenzene, which was reported to be more stable than those at the meta and para positions by Solà and co-workers^19^. As shown in Fig. 2, both **1a** and **1c** are much more stable than their nonaromatic analogues **2a** and **2c** thermodynamically. In sharp contrast, complexes **2b** and **2d** are thermodynamically more stable than metallaphosphabenzenes **1b** and **1d**. This could be one of the reasons why some metallapyridines have been isolated whereas metallaphosphabenzenes have never been synthesized. Apparently, the stability of iridaphosphabenzenes and iridapyridines relative to their nonaromatic analogues increases dramatically in comparison with rhodaphosphabenzenes and rhodapyridines due to more diffuse *d* orbitals, in line with previous results that 5*d* metallacycles are more stable than their 4*d* analogues^1^,^2^,^3^,^19^. These results are also applicable to ruthenaphosphabenzenes and osmaphosphabenzenes (Fig. 1B, **1a′–1d′** in Table 1). Especially for ruthenapyridine **1a′** and osmapyridine **1c′**, both of them are much more stable than their nonaromatic **2a′** and **2c′** by fixing the N-Cl bond with the value of 1.78 Å (the N-Cl bond length in **2a**) because **2a′** and **2c′** always rearranged to **1a′** and **1c′** by normal optimization.

What causes this significant difference between metallapyridines and metallaphosphabenzenes? It is well-known that the phosphorus atom has larger size difference in *s* and *p* valence orbitals than the nitrogen atom, leading to a lower tendency for hybridization to form multiple bonds^21^. Thus, the back donation from the *d* orbital of the metal center to the vacant *p* orbital of the phosphorus atom (Fig. 1C) will become weaker. Indeed, according to the NBO analysis of complexes **1c**–**1d** (Table 2), the results indicate that the M=P bond^38^,^39^,^40^,^41^ is highly polarized toward M^δ−^ – P^δ+^. For example, the nitrogen atom has contributed 1.27 electron population in the Ir=N double bond of **1c**. In sharp contrast, the contribution from the phosphorus atom is reduced by 0.41 electron in **1d** than that from the nitrogen atom in **1c**. Meanwhile, the NBO charge on the phosphorus atom becomes positive, which contrasts sharply with the negative charge on the nitrogen. Therefore, the reversed M=P bond polarization plays an important role in 1,2-migration in metallaphosphabenzenes. All these results indicate that the phosphorus atom is reluctant to participate in π bonding so that 1,2-migration in metallaphosphabenzenes could become thermodynamically favorable whereas such a migration in metallapyridines is unfavorable.

### Effect of ligands on the chloride migration in metallaphosphabenzenes

The effect of ligands has also been investigated to tune the stability of metallaphosphabenzenes. Our results indicated the ligands have a remarkable effect on the relative stability of metallaphosphabenzenes in comparison with the nonaromatic analogues (**1e**–**1h** and **2e**–**2h** in Fig. 2, **1e**′–**1h′** in Table 1). Specifically, when π-acceptor ligand CO is introduced to replace the chloride, reaction energies (Gibbs free energies) for the formation of **2f** and **2h** from **1f** and **1h** are −23.0 and −14.9 kcal mol^−1^, respectively, indicating that metallaphosphabenzenes prefer π-donor ligands. When the chloride is replaced by one ligand PH_3_, the instability of **1e** and **1g** is increased slightly in comparison with **1b** and **1d** in Fig. 2. This is understandable because the M=P double bond in metallaphosphabenzenes is strongly polarized toward M^δ−^ – P^δ+^, indicating that the phosphorus atom is highly electron-deficient. Therefore, π-acceptor ligand CO can decrease the electron density of the metal center, thus weakening the bonding between the metal center and metal-bonded phosphorus. In other words, the M=P double bond will become weaker and the polarization will be enhanced, leading to relatively high stability of nonaromatic **2f** and **2h**. The stabilizing effect of ligands in metallaphosphabenzenes increases in the order CO < PH_3_ < Cl. Therefore, π-donor and π-acceptor ligands are suggested for the synthesis of metallaphosphabenzenes and nonaromatic analogues, respectively.

### Effect of aromaticity on the chloride migration

**Figure 3:**
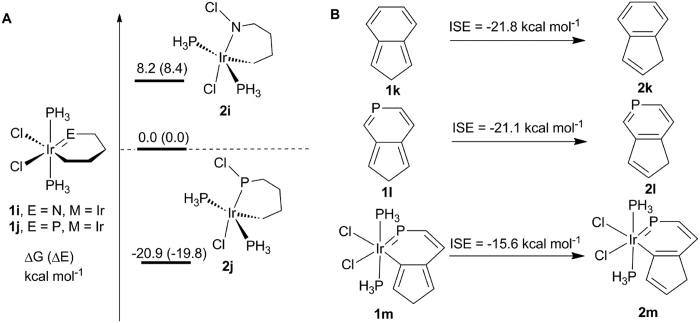
(**A**) Energy profiles calculated for the corresponding chloride migration of nonaromatic complexes. (**B**) Indene–isoindene ISE evaluations of the aromaticity of **1k**–**2m**.

The aromaticity effect has also been examined in metallaphosphabenzenes with similar migration of nonaromatic cyclic complexes by changing the M=E double bond to M-E single bond (Fig. 3A). Apparently, the 1,2-migration in nonaromatic cycles become more favorable thermodynamically. The reaction energy (ΔG) from **1i** to **2i** is computed to be 8.2 kcal mol^−1^ whereas that from **1j** to **2j** becomes −20.9 kcal mol^−1^. It is understandable because when aromaticity in the reactants is lost, such a chloride migration should become thermodynamically more favorable. The contribution from aromaticity in iridaphosphabenzene **1d** is thus evaluated quantitatively (−13.8 kcal mol^−1^) by computing the energy difference between the reaction from **1d** to **2d** and that from **1j** to **2j**.

To gain an insight into the aromaticity in metallaphosphabenzenes, we employed the “isomerization stabilization energy” (ISE) method, a convenient tool to evaluate the magnitude of the aromaticity in the ground state and the lowest triplet state^25^,^28^,^42^,^43^. The indene-isoindene ISE approach is homodesmotic and has the advantage that all carbon atoms in the six-membered ring are *sp*^2^-hybridized in both the reactants and products. As shown in Fig. 3B, benzene and phosphabenzene have comparable ISE values (−21.8 and −21.1 kcal mol^−1^, respectively). Nevertheless, the ISE value of iridaphosphabenzene is just 71.6% of that in benzene, indicating the aromaticity in metallaphosphabenzenes is fairly reduced. In addition, the ISE value (−15.6 kcal mol^−1^) of iridaphosphabenzene is comparable to the aromaticity contribution (−13.8 kcal mol^−1^), indicating the reliability of our calculations.

### 1,2-Migration of hydride and methyl groups in metallaphosphabenzenes

**Figure 4:**
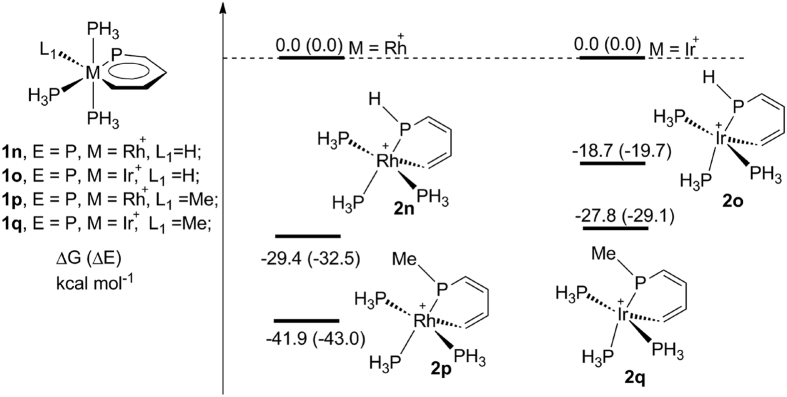
Thermodynamic stabilities of metallaaromatics (**1n**–**1q**) compared with its nonaromatic analogues (**2n**–**2q**) formed by hydride and methyl migration.

To examine the scope of our findings, we investigated the 1,2-migration of hydride and methyl groups (Fig. 4, and **1n′**–**1q′** in Table 1). The negative values indicate that these migrations are also feasible. The results in Fig. 4 show that the thermodynamic stability of metallaphosphabenzenes relative to its nonaromatic analogues with the hydride and methyl groups are higher than those with the chloride ligand, which could be mainly attributed to the higher bond strength of P-C and P-H bond than that of P-Cl in the products.

### Kinetics of chloride, hydride and methyl migration

**Figure 5:**
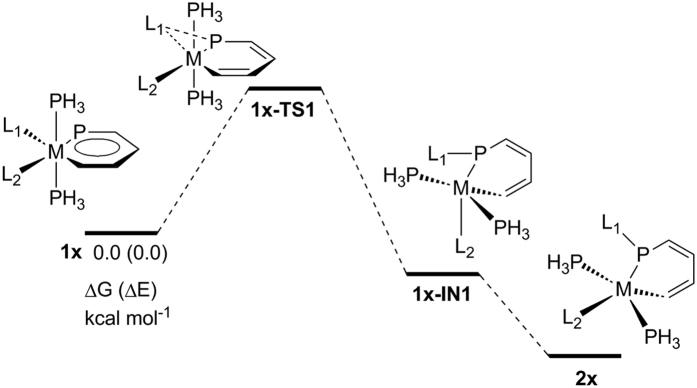
Energy profiles calculated for the 1,2-migration of metallaaromatics to its nonaromatic analogues.

To have a deeper understanding of the reaction mechanisms, we have examined the kinetics of 1,2-migration from metallaaromatics to its nonaromatic analogues (Fig. 5 and Table 3). The calculations show that reaction barriers are particularly low. All these values are lower than 24.2 kcal mol^–1^ at 25 °C according to the Eyring equation^44^. In addition, iridaphosphabenzene complexes have relative higher reaction barriers for 1,2-migration than rhodaphosphabenzene analogues. It could be due to the second-row transition metal rhodium has less diffuse *d* orbitals in comparison with iridium. Therefore, the Rh=P bond becomes relatively weak as evidenced by the Wiberg bond index (WBI) (1.00 and 1.07 for the Rh=P bond and Ir=P bond in **1b** and **1d**, respectively). The lowest barrier is found when the ligand is CO. As a strong π acceptor ligand, CO can reduce the interaction between the metal center and the phosphorus atom, thus weakening the metal-phosphorus bonds in metallaphosphabenzenes^41^. In addition, as shown in Table 3, the reaction barriers for the migration of hydride and methyl are a little higher than that of chloride. A plausible explanation for these general observations is clarified below. The results could be understandable because the Rh-Cl in **1e** has a weaker bond strength (WBI: 0.62) than Rh-H in **1n** (WBI: 0.65) and Rh-C in **1p** (WBI: 0.66), respectively. In addition, it is known that the M=P bond is highly polarized in metallaphosphabenzenes, and the ligand with higher electronegativity could promote the migration. Hence, a lower barrier for migration of chloride is expected in the reaction. In sharp contrast, all the metallapyridines are thermodynamically more stable than their nonaromatic analogues (Supplementary Fig. S1 and Table S1).

## Discussion

The 1,2-migration on a series of metallaphosphabenzenes and metallapyridines have been studied thoroughly by DFT calculations. The effects of metal centers, ligands, aromaticity, and migration groups were examined systematically. Our results reveal that due to the reluctance of phosphorus to participate in π bonding, such a migration in metallapyridines is thermodynamically unfavorable whereas it becomes feasible in metallaphosphabenzenes, which could be the origin of challenge of realizing metallaphosphabenzenes. The 4*d* transition metals and π-acceptor ligands have the tendency to form nonaromatic analogues rather than metallaphosphabenzenes whereas π-donor ligands and the 5*d* transition metals can stabilize metallaphosphabenzenes. In addition, the chloride migration has relatively lower reaction barrier due to the weaker bond strength of M-L_1_ in metallaphosphabenzene in comparison with hydride and methyl migrations. All these findings could be useful for synthetic chemists to realize the first metallaphosphabenzene.

## Methods

### Computational details

The M05^45^ level of density functional theory was applied to optimize all of the structures studied in the gas phase. Frequency calculations at the same level of theory have also been performed to identify all stationary points as minima (zero imaginary frequency) or transition states (one imaginary frequency). Calculations of intrinsic reaction coordinates (IRC)^46^,^47^ were also carried out on transition states to ensure that such structures are indeed connecting two minima. The LanL2DZ basis set^48^ was employed to describe Ru, Os, Rh, Ir, P and Cl whereas the 6–31G(d) basis set^49^ was used for all other atoms. Polarization functions were added for P (ξ(d) = 0.340), Cl (ξ(d) = 0.514), Ru (ξ(f) = 1.235), Os (ξ(f) = 0.886), Rh (ξ(f) = 1.350), and Ir (ξ (f) = 0.938)^50^,^51^. In order to examine the effect of function for these complexes, the density functional M06L^52^ has been used with basis sets unchanged. The relative Gibbs free energies of **2b** to **1b**, **2d** to **1d** are −13.1 and −4.1 kcal mol^−1^, respectively, which are comparable to those (−18.9 and −7.1 kcal mol^−1^) at the M05/6–31G(d) level, indicating that the functional dependence is small. To examine the effect of basis sets, we employed a larger 6-311 + G(d) basis set^53^ to optimize the complexes **1a**–**1d**, **2a**–**2d** (Fig. 2). The additional calculations show that the basis set dependence is small. For example, using the 6–31G(d) basis set in the gas phase, the relative free energies of **2a**–**2d** to **1a**–**b** are 0.0, 11.4, −18.9, 26.7 and −7.1 kcal mol^−1^, respectively. Using the larger 6-311 + G(d) basis set, the relative free energies are 0.0, 9.6, −20.1, 24.6 and −8.3 kcal mol^−1^, respectively. To examine the solvent effect^54^,^55^, we optimized the structures (**1b**, **1d**, **2b** and **2d**) using the PCM model^56^ with benzene as the solvent. The additional calculations show that the solvent effect is small. For example, the relative free energies of **2b** to **1b**, **2d** to **1d** are −16.1 and −5.0 kcal mol^−1^ (−18.9 and −7.1 kcal mol^−1^ in Fig. 2) when the solvent effect is included. Moreover, the solvent effect is also small for the transition states. For instance, when the solvent effect is included, the reaction barriers of **1e**–**TS1** and **1f**–**TS1** become 8.7 and 5.8 kcal mol^−1^, which are close to those in Table 3 (6.5 and 5.9 kcal mol^−1^, respectively). In addition, the polar solvents (ethanol and DMSO) are also been taken into account (see Supplementary Table S2), and the results for the polar solvents suggest that the solvent effect is also small. In order to examine the ligand effect of the simplified PH_3_, we use PMe_3_ to replace PH_3_. The results show that the ligand effect is small. For instance, using the ligand PMe_3_, the relative free energy of **2b** to **1b** is −14.0 kcal mol^−1^, which is close to −18.9 kcal mol^−1^ with the PH_3_ ligand in Fig. 2. Calculations on complexes **1a**–**1h** indicate that all of them in the singlet ground state are lower in energy than those in the lowest triplet state (Supplementary Table S3), indicating all of them have the close-shell singlet ground state. In addition, the thermodynamics of 1,2 migration on complexes **1a**–**1h** in the lowest triplet state are also examined and the results are similar to those in Fig. 2. Specifically, the relative Gibbs free energies of **2a**–**2h** to **1a**–**1h** are 14.6, −17.1, 40.8, −5.0, −19.3, −30.2, −10.1 and −16.3 kcal mol^−1^, respectively. The natural bond orbital (NBO, Version 3.1) was also used to obtain Wiberg bond indices (bond orders)^57^. All calculations were carried out with the Gaussian 03 package^58^ except the M06L calculations, which were performed by Gaussian 09 package^59^. All the relative Gibbs free energies calculated at 298 K and electronic energies (in parentheses) are given in kcal mol^−1^.

## Additional Information

**How to cite this article**: Wu, J. *et al*. Probing the Origin of Challenge of Realizing Metallaphosphabenzenes: Unfavorable 1,2-Migration in Metallapyridines Becomes Feasible in Metallaphosphabenzenes. *Sci. Rep*. **6**, 28543; doi: 10.1038/srep28543 (2016).

## Supplementary Material

Supplementary Information

## Figures and Tables

**Table 1:** Calculated reaction energies for the 1,2-migration of metallaaromatics to its nonaromatic analogues based on Fig. 1B.

Complex(1′)	M	E	L_1_	L_2_	PR(2′)
**1a**′	Ru^−^	N	Cl	Cl	35.1 (36.7)
**1b**′	Ru^−^	P	Cl	Cl	−1.4 (−0.4)
**1c**′	Os^−^	N	Cl	Cl	54.6 (56.7)
**1d**′	Os^−^	P	Cl	Cl	10.4 (10.5)
**1e**′	Ru	P	Cl	PH_3_	−2.6 (−2.4)
**1f**′	Ru	P	Cl	CO	−8.7 (−9.7)
**1g**′	Os	P	Cl	PH_3_	7.8 (7.1)
**1h**′	Os	P	Cl	CO	1.8 (1.3)
**1n**′	Ru	P	H	PH_3_	−18.9 (−20.3)
**1o**′	Os	P	H	PH_3_	−5.4 (−7.2)
**1p**′	Ru	P	Me	PH_3_	−26.9 (−28.9)
**1q**′	Os	P	Me	PH_3_	−13.6 (−14.6)

The relative Gibbs free energies at 298 K and electronic energies (in parentheses) are given in kcal mol^−1^.

**Table 2:** The charges and electron populations of M=E bonds (E=N, or P) in **1a**–**1d**.

	Charge (M)	Charge (E)	Electron population (E)
**1a**	−0.69	−0.32	1.23
**1b**	−1.14	+0.88	0.83
**1c**	−0.57	−0.37	1.27
**1d**	−1.09	+0.82	0.86

**Table 3:** Calculated reaction energies and barriers for the 1,2-migration of metallaaromatics to its nonaromatic analogues based on Fig. 5.

1X			1X–TS1	1X–IN1	2X
M = Os	L_2_ = PH_3_	L_1_ = Cl	22.2 (20.8)	18.7 (18.6)	7.8 (7.1)
M = Os	L_2_ = CO	L_1_ = Cl	14.0 (12.5)	12.8 (11.5)	1.8 (1.3)
M = Os	L_2_ = PH_3_	L_1_ = H	17.3 (16.7)	0.7 (−0.7)	−5.4 (−7.2)
M = Ru	L_2_ = PH_3_	L_1_ = Cl	14.6 (14.0)	5.9 (6.3)	−2.6 (−2.4)
M = Ru	L_2_ = CO	L_1_ = Cl	9.5 (7.8)	−1.5 (−1.3)	−8.7 (−9.7)
M = Ru	L_2_ = PH_3_	L_1_ = H	10.9 (11.3)	−14.6 (−14.6)	−18.9 (−20.3)
M = Rh^+^	L_2_ = PH_3_	L_1_ = Cl	6.5 (6.1)	−11.2 (−9.0)	−19.2 (−20.8)
M = Rh^+^	L_2_ = CO	L_1_ = Cl	5.9 (4.7)	−11.1 (−10.6)	−23.0 (−25.0)
M = Rh^+^	L_2_ = PH_3_	L_1_ = H	8.8 (8.2)	−22.3 (−23.2)	−29.4 (−32.5)
M = Rh^+^	L_2_ = PH_3_	L_1_ = Me	8.4 (8.9)	−33.1(−32.4)	−41.9(−43.0)
M = Ir^+^	L_2_ = PH_3_	L_1_ = Cl	12.3 (10.5)	4.2 (4.2)	−8.3 (−10.2)
M = Ir^+^	L_2_ = CO	L_1_ = Cl	8.1 (6.5)	0.9 (0.4)	−14.9 (−16.4)
M = Ir^+^	L_2_ = PH_3_	L_1_ = H	14.2 (14.6)	−9.1 (−8.9)	−18.7 (−19.7)
M = Ir^+^	L_2_ = PH_3_	L_1_ = Me	14.5 (14.6)	−17.2 (−15.9)	−27.8 (−29.1)

The relative Gibbs free energies at 298 K and electronic energies (in parentheses) are given in kcal mol^−1^.
