# Supplementary material for: Probing the Origin of Challenge of Realizing Metallaphosphabenzenes: Unfavorable 1,2-Migration in Metallapyridines Becomes Feasible in Metallaphosphabenzenes
Source: Sci Rep. 2016 Jun 24;6:28543. doi: 10.1038/srep28543 (PMC4919789; doi:10.1038/srep28543)
Supplement: Supplementary Information [file srep28543-s1.doc]

**Supplementary Information**

**Probing the Origin of Challenge of Realizing Metallaphosphabenzenes: Unfavorable 1,2-Migration in Matallapyridines Becomes Feasible in Metallaphosphabenzenes**

Jingjing Wu^1^, Yulei Hao^1^ and Jun Zhu^1^*

^1^State Key Laboratory of Physical Chemistry of Solid Surfaces, Collaborative Innovation Center of Chemistry for Energy Materials (iChEM), Fujian Provincial Key Laboratory of Theoretical and Computational Chemistry, Department of Chemistry, College of Chemistry and Chemical Engineering, Xiamen University, Xiamen 361005

*Corresponding author: Jun Zhu

Address: 422#, Siming South Road, Siming District, Xiamen, Fujian, China

Email: jun.zhu@xmu.edu.cn

**Contents**

1. **Supplementary Figures S1**
2. **Supplementary Table S1-S3**
3. **Cartesian coordinates**

**Supplementary Figure S1**. Energy profiles calculated for the 1,2-migration of metallaaromatics to its nonaromatic analogues.

**Supplementary Table S1.** Calculated reaction energies for the 1,2-migration of metallaaromatics to their nonaromatic analogues. The Gibbs free energies and relative electronic energies at 298 K (in parentheses) are given in kcal mol^-1^.

|  | **PR** |
| --- | --- |
| M = Os^-^, L_1_ = Cl, L_2_ = Cl  M = Os, L_1_ = Cl, L_2_ = PH_3_  M = Os, L_1_ = Cl, L_2_ = CO  M = Ru^-^, L_1_ = Cl, L_2_ = Cl  M = Ru, L_1_ = Cl, L_2_ = PH_3_  M = Ru, L_1_ = Cl, L_2_ = CO  M = Rh, L_1_ = Cl, L_2_ = Cl  M = Rh^+^, L_1_ = Cl, L_2_ = PH_3_  M = Rh^+^, L_1_ = Cl, L_2_ = CO  M = Ir, L_1_ = Cl, L_2_ = Cl  M = Ir^+^, L_1_ = Cl, L_2_ = PH_3_  M = Ir^+^, L_1_ = Cl, L_2_ = CO | 56.1 (57.2) |
|  | 47.6 (48.3) |
|  | 55.9 (56.1) |
|  | 34.6 (37.1) |
|  | 34.6 (34.2) |
|  | 24.4 (24.5) |
|  | 11.4 (11.9) |
|  | 16.4 (17.7) |
|  | 11.9 (14.5) |
|  | 26.7 (27.3) |
|  | 31.2 (31.1) |
|  | 31.7 (33.7) |

**Supplementary Table S2.** The reaction barrier for the 1,2-migration of metallaaromatics to its nonaromatic analogues based on Figure 5. The relative Gibbs free energies at 298 K and electronic energies (in parentheses) are given in kcal mol^-1^.

|  | gas | DMSO | ethanol |
| --- | --- | --- | --- |
| **1e-TS1** | 6.5 | 9.6 | 8.9 |
| **1f-TS1** | 5.9 | 9.1 | 9.9 |

**Supplementary Table S3.** The relative free energy difference (ΔG_S-T_) between the S_0_ and T_1_ states of complexes **1a**-**1h**. The energies are given in kcal mol^-1^.

|  | **1a** | **1b** | **1c** | **1d** | **1e** | **1f** | **1g** | **1h** |
| --- | --- | --- | --- | --- | --- | --- | --- | --- |
| ΔG_S-T_ | 0.7 | 19.7 | 1.8 | 21.5 | 21.1 | 25.6 | 23.7 | 23.5 |

**Cartesian coordinates and electronic energies for all the species calculated in this study**

**1a**

E = -365.2712716 a.u.

C -2.74905000 -0.06831300 1.18734200

C -1.35290700 -0.07081200 1.37102100

H -3.39624700 -0.11526000 2.06166800

H -0.99455300 -0.12142300 2.40298700

C -2.58136900 0.06384900 -1.29132300

C -3.32932500 -0.00601900 -0.05516800

H -3.19272600 0.10979300 -2.20334500

H -4.41563300 -0.00631400 -0.12968300

Cl 1.67484100 -0.10241900 1.90061100

Cl 1.86668600 0.08640000 -1.68153000

P 0.52690900 -2.27773200 -0.23264000

H 0.27047500 -2.82095700 -1.52222900

H 1.90578800 -2.53710900 -0.04342700

H -0.06847900 -3.27723900 0.59955900

P 0.51995600 2.29612800 -0.03023000

H 1.89037700 2.54883200 0.22154300

H 0.29806900 2.93588700 -1.28099600

H -0.10667100 3.22619300 0.85857100

N -1.30694600 0.07969600 -1.44870100

Rh 0.02500400 -0.00035800 0.04719300

**1a-T1**

E_T1_ = -365.266178 a.u.

C 2.74572800 0.03291300 1.12363800

C 1.39058500 0.05186800 1.35109000

H 3.40328200 0.06681200 1.99256900

H 1.02940800 0.10018100 2.37681700

C 2.60873600 -0.08090700 -1.33829700

C 3.37356400 -0.02819900 -0.13658400

H 3.11559900 -0.12996500 -2.30640200

H 4.45520200 -0.03716300 -0.21830800

Cl -1.62862200 0.11842200 1.85140500

Cl -2.00127200 -0.08844200 -1.64722700

P -0.37913800 2.34288200 -0.12754500

H 0.07741400 3.01694900 -1.29878400

H -1.73902100 2.74555800 -0.08613200

H 0.19148400 3.17608300 0.88124600

P -0.42559400 -2.33772300 0.06790700

H -1.78184700 -2.70789600 0.25991800

H -0.08574300 -3.08997000 -1.09408600

H 0.21967800 -3.12101000 1.07336500

N 1.32744300 -0.07451100 -1.33372100

Rh -0.11355700 0.00135300 -0.01821500

**2a**

E = -365.252312697 a.u.

C 0.86854800 -0.80969300 2.35853800

C -0.27928800 -0.42705100 1.76846100

H 0.79438400 -1.06422000 3.41771200

H -1.25183300 -0.42507200 2.25818300

C 2.46940600 -0.64908600 0.45901400

C 2.18434700 -0.88465700 1.77370400

H 3.50531700 -0.67107700 0.12564900

H 3.00996800 -1.18092900 2.41228700

Cl -2.75049000 0.70146200 -0.17472200

P 0.11342100 2.22170300 0.55826900

H 0.12283000 3.30137000 -0.37645200

H -0.74006200 2.77426700 1.55329800

H 1.38955800 2.41513500 1.16532900

P -1.11844600 -2.17899600 -0.62762400

H -1.71882900 -2.48016500 -1.88800700

H -0.13141200 -3.20950800 -0.58760800

H -2.10356800 -2.74552700 0.23270000

N 1.52945700 -0.50150700 -0.51849000

Cl 2.12305700 0.48992300 -1.86646000

Rh -0.42886400 0.05611200 -0.15781200

**2a-T1**

E_T1_ = -365.241308150 a.u.

C 2.39383700 -1.67771300 0.30753100

C 1.02470900 -1.85081200 0.33763100

H 3.02320300 -2.55478600 0.46808700

H 0.68145600 -2.87428200 0.52582700

C 2.54091800 0.80446800 -0.13195900

C 3.09413600 -0.47494200 0.09254200

H 3.22769400 1.63789000 -0.27523300

H 4.17939000 -0.50864300 0.09876300

Cl -2.58855300 0.65438200 -0.14129100

P -0.69266300 -0.06667100 2.41039900

H -0.75651700 1.31986700 2.73769100

H -1.89588000 -0.49325100 3.04627600

H 0.23879200 -0.48096200 3.41641800

P -0.64077100 -0.89186200 -2.25186900

H -0.69591600 0.29840900 -3.03600000

H 0.31006700 -1.62168700 -3.03555400

H -1.83169800 -1.51660300 -2.72585700

N 1.24594800 1.02662600 -0.17081300

Cl 0.81506700 2.67930300 -0.44976600

Rh -0.43051200 -0.52206700 0.08912900

**1b**

E = -317.083821 a.u.

C 2.47574500 1.13757500 1.21502000

C 1.09886600 1.06196500 1.14913500

H 2.91802300 1.82441500 1.93633100

H 0.55235000 1.71145300 1.83772100

C 3.05548500 -0.55059200 -0.54659800

C 3.37273800 0.39594900 0.43071000

H 3.89939000 -1.02798700 -1.04531200

H 4.43233500 0.57981000 0.61289000

Cl -2.03769300 1.26667800 1.21221900

Cl -1.71883900 -1.32117400 -1.38878700

P -0.57011700 1.75725700 -1.48971000

H 0.08247400 1.80826400 -2.76041100

H -1.93354900 1.76690000 -1.87342800

H -0.36639700 3.09581700 -1.04872000

P -0.68094700 -1.70738800 1.53479700

H -2.08471400 -1.78712500 1.71065500

H -0.34461200 -3.05113700 1.20320500

H -0.22397200 -1.65903200 2.88734200

P 1.48802200 -1.09236000 -1.08368300

Rh -0.14759200 0.02295700 0.03623800

**1b-T1**

E_T1_ = -317.048454449 a.u.

C -2.48974600 -0.56936400 1.47714700

C -1.12967900 -0.59337000 1.38701700

H -2.91683200 -0.82705600 2.44702000

H -0.58919300 -0.89656800 2.28209400

C -3.10351000 -0.10205700 -0.88863900

C -3.43294400 -0.27764500 0.45107100

H -3.90303800 0.04550600 -1.61268100

H -4.48467000 -0.23585500 0.72680500

Cl 1.76967600 0.11547500 1.91991400

Cl 2.00844000 0.69231200 -1.61211800

P 1.14672000 -2.21547600 -0.28590800

H 0.90274400 -2.94310700 -1.48924000

H 2.56578500 -2.24985000 -0.23558100

H 0.81496900 -3.21714800 0.67534400

P -0.12528600 2.26946500 0.29988300

H 1.03746800 2.96405800 0.72706900

H -0.53299700 3.05944700 -0.81279100

H -1.07858300 2.68458800 1.27702500

Rh 0.26684100 -0.02138200 -0.02355800

P -1.49584700 -0.18062900 -1.52844500

**2b**

E = -317.114327355 a.u.

C 1.89172800 -2.24499500 0.51851700

C 0.55637800 -2.00807300 0.54209800

H 2.25769200 -3.21836100 0.84781400

H -0.08383500 -2.86634600 0.77855500

C 2.64440500 -0.13682900 -0.56219500

C 2.89585000 -1.29975600 0.09349400

H 3.45977800 0.53540900 -0.81654300

H 3.93385000 -1.55382000 0.30782100

Cl -2.39380900 1.25424700 0.58528500

P 0.52830000 0.79152500 2.00728100

H 1.83322400 0.39943300 2.43115500

H 0.71282500 2.19097700 1.83479700

H -0.15181500 0.78442300 3.26089300

P -2.02347700 -1.28846300 -1.36512100

H -2.39656600 -0.37796300 -2.39405900

H -1.68486500 -2.41804600 -2.17765100

H -3.32450300 -1.70068900 -0.94963900

Cl 1.19972700 2.37633700 -1.20976000

P 1.02831700 0.24980100 -1.28055400

Rh -0.55963700 -0.34777700 0.30038600

**2b-T1**

E_T1_ = -317.074184958 a.u.

C -1.47027000 -2.59031600 -0.00208000

C -0.18650900 -2.12729800 -0.00190200

H -1.61121100 -3.67242500 -0.00292100

H 0.58853900 -2.90369300 -0.00282400

C -2.81330700 -0.45135100 -0.00080700

C -2.68338700 -1.83050400 -0.00145400

H -3.80329600 -0.00609300 -0.00045400

H -3.60924200 -2.40294400 -0.00153300

Cl 2.51776100 1.36624600 0.00182600

P 0.99127500 -0.28790600 -2.36961600

H 0.09151200 -0.97374600 -3.24380000

H 0.95723700 1.00588300 -2.96420500

H 2.22504600 -0.75179300 -2.91855700

P 0.98787400 -0.29113500 2.37041900

H 0.95230200 1.00177500 2.96692400

H 0.08738800 -0.97865700 3.24256000

H 2.22119100 -0.75502700 2.92041200

Cl -2.30360900 2.53976000 0.00044400

P -1.43500200 0.61985000 -0.00060000

Rh 0.73374600 -0.32401500 0.00000500

**1c**

E = -360.4657251 a.u.

C -2.67019300 -0.49606900 1.16616900

C -1.26559800 -0.56595500 1.31237400

H -3.30253400 -0.70523000 2.02703700

H -0.90896400 -0.93814700 2.27889900

C -2.54094900 -0.16839300 -1.29839500

C -3.26695900 -0.29680500 -0.05615800

H -3.15909600 -0.15635100 -2.20510200

H -4.35222800 -0.35626200 -0.12308500

Cl 1.72840800 0.05380600 1.85190400

Cl 1.76531800 0.75594300 -1.63482300

P 1.05844000 -2.13484800 -0.41779700

H 0.78739600 -2.73767600 -1.67994700

H 2.47091200 -2.03007300 -0.41903100

H 0.84410400 -3.23792400 0.46174200

P -0.17630400 2.28950900 0.22431600

H 1.03834100 2.91924800 0.59419100

H -0.54876300 2.97784900 -0.96235800

H -1.09340500 2.85075300 1.16634100

N -1.26257400 -0.18210000 -1.46305100

Ir 0.03765000 -0.05498500 0.02039700

**1c-T1**

E_T1_ = -360.461131896 a.u.

C 2.74643700 0.09650000 1.12800400

C 1.38844200 0.16389200 1.35056800

H 3.40769900 0.20125300 1.98718600

H 1.05163700 0.31857400 2.37474000

C 2.58867600 -0.24845900 -1.31131000

C 3.36101700 -0.09833400 -0.12457200

H 3.08932100 -0.39532300 -2.27197600

H 4.44217400 -0.13718100 -0.20985000

Cl -1.64453500 0.33729500 1.82167700

Cl -2.00521300 -0.24365800 -1.61167900

P -0.25883600 2.34183100 -0.30092600

H 0.45374500 2.95407200 -1.37489100

H -1.58558500 2.78500400 -0.54681000

H 0.13410000 3.17429500 0.78500100

P -0.41118400 -2.32560200 0.25618300

H -1.74862000 -2.65740000 0.59113200

H -0.17480000 -3.14939300 -0.88092300

H 0.32091600 -3.03253500 1.25720100

Ir -0.08982500 0.00196900 -0.02238200

N 1.30207900 -0.21855100 -1.30604900

**2c**

E = -360.422162996 a.u.

C -0.91921100 1.18154100 2.25407000

C 0.22203900 0.70624900 1.71260100

H -0.84831700 1.57644400 3.26852900

H 1.17897600 0.76628600 2.23242000

C -2.51668700 0.77713200 0.38032100

C -2.22546900 1.20929600 1.64190200

H -3.54451200 0.81490900 0.02511400

H -3.04077600 1.65362400 2.20431100

Cl 2.70437200 -0.67697700 -0.04560900

P -0.20131800 -2.14703800 0.81205000

H -0.17652900 -3.31943300 -0.00146000

H 0.61732600 -2.58190200 1.89211400

H -1.49905300 -2.26796100 1.38896400

P 0.97578400 2.08501700 -0.89235400

H 1.48780800 2.23073000 -2.21722100

H -0.04617400 3.07946300 -0.91061500

H 1.99589400 2.76321100 -0.16499400

N -1.58338700 0.40587600 -0.54814800

Cl -2.23804900 -0.81453900 -1.68551400

Ir 0.36429300 -0.05864600 -0.11921800

**2c-T1**

E_T1_ = -360.3924157 a.u.

C -1.09223000 -1.53201700 2.17619400

C 0.09402500 -1.11190700 1.60603500

H -1.04319200 -2.15363400 3.07111600

H 1.02221800 -1.42528600 2.08820300

C -2.64317700 -0.42726900 0.58322100

C -2.37931500 -1.22047900 1.69889800

H -3.67224800 -0.23520700 0.29598600

H -3.24883000 -1.60697800 2.22055000

Cl 2.73642400 -0.10685000 0.19651000

P 0.61833500 -1.67251700 -1.74630800

H 1.27644200 -1.43801200 -2.99748500

H 1.41334100 -2.77794500 -1.32272200

H -0.50749400 -2.39756100 -2.25303400

P 0.62340400 2.34858000 0.61498500

H 1.26448900 3.32950200 -0.21153100

H -0.50477400 3.13429900 1.01114300

H 1.43547100 2.52309700 1.77410300

N -1.68530600 0.12165000 -0.16171000

Cl -2.31057100 1.08482900 -1.48715600

Ir 0.31974400 0.01532000 -0.00025500

**1d**

E = -312.273532422 a.u.

C -2.45613700 -1.28163300 1.10616300

C -1.07301800 -1.21007900 1.01984800

H -2.88448700 -1.98714900 1.81693800

H -0.54593800 -1.91367600 1.67211400

C -3.06836100 0.34405700 -0.70464500

C -3.36372900 -0.55878000 0.32164100

H -3.92268900 0.76219500 -1.23658600

H -4.41970400 -0.75686900 0.51119900

Cl 2.00744300 -0.95705700 1.45792700

Cl 1.71283200 1.50800700 -1.18718400

P 1.03671800 -1.70516500 -1.39332600

H 0.55684400 -1.85851100 -2.72960100

H 2.42093000 -1.49201700 -1.60601100

H 0.99365400 -3.05488500 -0.94262800

P 0.09156500 1.81446000 1.45303000

H 1.37143000 2.04269200 2.01963100

H -0.23130800 3.07990500 0.89208600

H -0.74488400 1.80750800 2.61091300

P -1.50879300 0.82070800 -1.32742500

Ir 0.12515100 -0.04814000 0.01230000

**1d-T1**

E_T1_ = -312.2356201 a.u.

C 2.53646900 0.43182900 1.49904100

C 1.17039700 0.41675100 1.43583100

H 2.97214400 0.71028000 2.45853300

H 0.66764000 0.68877300 2.36359300

C 3.15401500 -0.25621000 -0.82512000

C 3.47378800 0.13158900 0.47293600

H 3.95975800 -0.45981100 -1.52708400

H 4.53009400 0.20995900 0.72318700

Cl -1.85055200 0.60180500 1.73243400

Cl -2.03450500 -0.52314600 -1.60338000

P -0.37956900 2.28004100 -0.59911700

H 0.37433900 2.78863800 -1.69837000

H -1.69419000 2.68472400 -0.95477500

H -0.04536700 3.22594100 0.41114400

P -0.43257100 -2.26248900 0.65168900

H -1.72434300 -2.58403300 1.14921400

H -0.24592800 -3.26839700 -0.33784700

H 0.40354400 -2.75664400 1.69650900

P 1.54746300 -0.45364700 -1.43843600

Ir -0.21025400 -0.00492100 -0.01540500

**2d**

E = -312.2855659 a.u.

C 1.76543400 -2.27640900 0.74343700

C 0.46145300 -1.89906600 0.75904700

H 2.04265200 -3.22061200 1.21189000

H -0.23335900 -2.64449000 1.17016300

C 2.71257100 -0.43315900 -0.65350900

C 2.84105900 -1.53805800 0.12450700

H 3.60327800 0.04429200 -1.05660400

H 3.84762500 -1.92262700 0.29031800

Cl -2.30503800 1.45221900 0.29308000

P 0.63385700 1.00752700 1.87167000

H 2.05053700 0.89685800 1.96487400

H 0.45150200 2.41750100 1.85706200

H 0.26983700 0.71934000 3.22192200

P -1.87948400 -1.28072500 -1.38995200

H -2.15574100 -0.49701800 -2.54547100

H -1.47814800 -2.50343100 -2.01230200

H -3.20958800 -1.63033000 -1.01259300

Cl 1.60984600 2.28172200 -1.16744800

P 1.14279600 0.17869000 -1.32351100

Ir -0.50014200 -0.21868400 0.24105200

**2d-T1**

E_T1_ = -312.2400393 a.u.

C 1.47455300 -2.60059800 -0.00279400

C 0.20159700 -2.09529500 -0.00227700

H 1.58352900 -3.68562700 -0.00397700

H -0.57716800 -2.86924600 -0.00323400

C 2.88136400 -0.50105400 -0.00086600

C 2.70645400 -1.87684300 -0.00206600

H 3.88433500 -0.08555600 -0.00056100

H 3.61622400 -2.47528800 -0.00258200

Cl -2.31700100 1.54720200 0.00210600

P -0.83551800 -0.21989800 2.35338200

H 0.10286100 -0.92756400 3.16409600

H -0.78757000 1.06495600 2.96622800

H -2.05108700 -0.70553100 2.92352300

P -0.83788600 -0.21449700 -2.35328600

H -0.78657100 1.07143500 -2.96354600

H 0.09724400 -0.92340400 -3.16668600

H -2.05572400 -0.69497600 -2.92288400

Cl 2.48351200 2.52104400 0.00147900

P 1.54511400 0.61781500 0.00007700

Ir -0.61709400 -0.24984300 -0.00007600

**1e**

E = -310.2045143 a.u.

C 2.44049300 -0.17247200 1.75800900

C 1.06223500 -0.10611600 1.65976500

H 2.87579100 -0.31595200 2.74597200

H 0.55061000 -0.17342800 2.62450300

C 3.04699000 0.24508500 -0.64596300

C 3.34622700 -0.02255200 0.70070500

H 3.90105700 0.37912900 -1.30998000

H 4.40223600 -0.08224900 0.96132100

P -0.68664900 2.33802700 0.16535500

H -0.50913400 3.00057300 -1.07911300

H -2.04953200 2.63774000 0.43798000

H -0.02345400 3.23443400 1.05507900

P 0.07738600 -2.23248200 -0.60656400

H -1.05860600 -3.06331000 -0.39552200

H 0.26439300 -2.38904400 -2.00601600

H 1.12118200 -3.05208600 -0.08482100

P 1.51206600 0.48414200 -1.40374300

P -2.29970000 -0.55494100 1.46742500

H -2.34438600 -0.83255500 2.87136500

H -3.35506900 0.40201500 1.41005500

H -3.03360700 -1.68666400 1.00568100

Cl -1.85067200 0.06965500 -1.71297000

Rh -0.17116200 0.01272000 0.12692900

**1e-T1**

E_T1_ = -310.167505114 a.u.

C 2.46013000 0.10914200 1.65771900

C 1.10065200 0.16959900 1.56535500

H 2.87224700 0.10957300 2.66699300

H 0.58204100 0.27302400 2.51980300

C 3.15764700 0.22866800 -0.74360200

C 3.42916000 0.06804000 0.61317500

H 3.98674200 0.22802700 -1.44883700

H 4.46925500 -0.06454700 0.90023300

P -0.84448000 2.34774600 0.08484200

H -0.85512300 2.96981000 -1.19341500

H -2.14580900 2.69462000 0.55083000

H -0.04134900 3.25739100 0.83185000

P 0.34422600 -2.24252400 -0.36821300

H -0.41504800 -3.26108300 0.28093300

H 0.23055600 -2.65698500 -1.72286700

H 1.66737200 -2.64913600 -0.03940100

P 1.58225600 0.52492300 -1.38854900

P -2.06537900 -0.58432800 1.49956600

H -1.78030500 -0.68378700 2.89742100

H -3.20336800 0.26786100 1.51838900

H -2.68156900 -1.84349600 1.26321000

Cl -2.19251300 -0.22229100 -1.50260700

Rh -0.25661800 0.02217200 0.01219100

**1e-TS1**

E = -310.1947929 a.u.

C -2.00768700 0.05849100 -2.03015900

C -0.65286600 0.13081400 -1.87186700

H -2.40237000 0.04113900 -3.04533000

H -0.05095800 0.17078800 -2.78366400

C -2.85597300 0.19913600 0.37995200

C -2.99057800 0.03516300 -0.99508700

H -3.78055000 0.21102400 0.95686500

H -4.01829500 -0.04245200 -1.35046100

P 1.08062900 2.30702100 -0.22711300

H 1.22284700 2.93187500 1.04389200

H 2.36810300 2.56575500 -0.78480500

H 0.31472100 3.28510400 -0.92415600

P -0.52180400 -2.17174900 0.20475400

H 0.47710500 -3.15035000 0.47384200

H -1.38361100 -2.38218700 1.31381900

H -1.24901800 -2.81866900 -0.83746800

P -1.43316300 0.88182100 1.17162900

P 2.49723000 -0.88276300 -0.98818200

H 2.68004600 -1.20096100 -2.36965800

H 3.67557800 -0.09719900 -0.80738200

H 2.98976100 -2.10053400 -0.43074000

Cl 0.71885200 -0.27312000 2.36790800

Rh 0.30300900 0.05940300 -0.13383400

**1e-IN1**

E = -310.2188334 a.u.

C 1.03012800 -0.62229200 2.48734800

C -0.14259000 -0.33989900 1.88909200

H 0.97839500 -0.86429500 3.54973300

H -1.05398200 -0.26734100 2.48746000

C 2.67988800 -0.16062200 0.68087900

C 2.34643400 -0.70133000 1.87717800

H 3.68867800 -0.26718700 0.28820800

H 3.11419900 -1.22552800 2.44298700

P -1.31577700 2.22664900 0.55278100

H -1.21780400 3.20876800 -0.47637500

H -2.65718900 2.46998300 0.98127000

H -0.60021000 2.88815700 1.59153700

P 0.35055800 -2.13302000 -0.53105300

H -0.53483700 -3.13320000 -1.03648400

H 1.38442100 -2.23008300 -1.50411500

H 0.92365000 -2.84327700 0.55972300

P 1.58369200 1.03633800 -0.13138600

P -2.99704800 -0.81761700 -0.52484800

H -3.56660400 -1.56942400 0.54816100

H -4.10703300 0.05396500 -0.74736500

H -3.28306300 -1.74752100 -1.57095500

Cl 1.91654000 0.63894400 -2.17741600

Rh -0.56565200 0.02054600 -0.04860200

**2e**

E = -310.237732 a.u.

C 2.18683500 -1.98078000 0.16856600

C 0.85044500 -1.97309600 0.41722200

H 2.73617800 -2.90601700 0.34241400

H 0.39755900 -2.94301100 0.64777400

C 2.48995000 0.31106500 -0.78788100

C 2.97287300 -0.86583400 -0.29234900

H 3.17808100 1.11797500 -1.03084500

H 4.05431600 -0.97824300 -0.23346500

P 0.77485500 0.56829500 2.06330900

H 1.01865900 1.96996600 1.97566200

H 0.22219800 0.50089400 3.37777200

H 2.08422900 0.07196000 2.30820900

P -1.78325300 -1.78328600 -1.21596500

H -2.32306100 -1.11870100 -2.35659700

H -1.11020400 -2.85564800 -1.87105100

H -2.95523600 -2.47705700 -0.78549700

Cl 0.60155200 2.60386400 -1.11393100

P 0.78982000 0.51279100 -1.33057500

P -2.29486700 1.25071100 0.52082200

H -2.77716100 1.83358500 -0.68842700

H -3.55344400 0.88629300 1.08959400

H -2.09044600 2.45905300 1.25216500

Rh -0.49793300 -0.46672300 0.38470900

**2e-T1**

E_T1_ = -310.199063642 a.u.

C -1.58898600 -2.44969900 0.00141900

C -0.26455600 -2.13328500 0.00134300

H -1.83702100 -3.51141500 0.00206800

H 0.43682400 -2.97546100 0.00195200

C -2.75805000 -0.19094100 0.00011400

C -2.72958700 -1.57738200 0.00078800

H -3.71394500 0.32477300 -0.00018100

H -3.69897600 -2.06972200 0.00090900

P 0.77352200 -0.69084500 -2.38520400

H 1.26754200 0.30655600 -3.27990000

H 1.47969300 -1.82307500 -2.89059100

H -0.50307400 -0.92606000 -2.96860200

P 0.77468600 -0.68744900 2.38589500

H 1.26759500 0.31210300 3.27880300

H -0.50136600 -0.92352900 2.97014900

H 1.48264100 -1.81785100 2.89288900

Cl -1.91995500 2.75846300 -0.00083500

P -1.31469500 0.78163400 -0.00022500

P 2.29928700 1.57907300 -0.00143500

H 2.23761800 2.51663600 1.07319900

H 3.71194300 1.37186800 0.00037500

H 2.23986700 2.51310100 -1.07927200

Rh 0.77388800 -0.37378100 0.00011000

**1f**

E = -415.184674 a.u.

C -2.47077400 -0.00099900 1.71453200

C -1.08937500 -0.00105300 1.66925700

H -2.94659900 -0.00157100 2.69369200

H -0.61989900 -0.00182800 2.65608600

C -2.97160300 0.00032400 -0.74862300

C -3.33079600 -0.00036000 0.61036700

H -3.79532100 0.00074700 -1.46292000

H -4.39729500 -0.00041600 0.83177500

P 0.38562600 -2.35070900 -0.20391100

H 0.19928600 -2.74736400 -1.55450500

H 1.68082400 -2.86840400 0.06775000

H -0.45042600 -3.27363900 0.49035700

P 0.38502100 2.35087100 -0.20219600

H 1.68003500 2.86873800 0.07005400

H 0.19877800 2.74847700 -1.55253400

H -0.45137200 3.27304400 0.49267200

P -1.40915300 0.00063400 -1.48167300

Cl 1.91327000 0.00073400 -1.63413200

C 1.94932500 -0.00026600 1.49848600

O 2.93556000 -0.00033700 2.06513100

Rh 0.22108600 -0.00012000 0.18620800

**1f-T1**

E_T1_ = -415.142717954 a.u.

C -2.46075800 -0.30706900 1.61217900

C -1.09704800 -0.32857600 1.57534700

H -2.91225800 -0.40267900 2.60010600

H -0.61491100 -0.50876000 2.53599800

C -3.06132100 -0.27506300 -0.81510700

C -3.38799900 -0.18847200 0.53549300

H -3.85504500 -0.20762600 -1.55702800

H -4.43607600 -0.04833400 0.78798100

P 1.17284100 -2.24972000 0.06784200

H 1.14306600 -2.85137100 -1.21823200

H 2.54621100 -2.38005000 0.41272000

H 0.57011600 -3.25388900 0.87982700

P -0.40104800 2.24706300 -0.30308300

H 0.39894700 3.26665900 0.28568000

H -0.38140500 2.62830800 -1.67061200

H -1.70655800 2.62801600 0.11461200

P -1.46435400 -0.62494200 -1.38291600

Cl 2.11052200 0.37561600 -1.55005400

C 1.66321500 0.56519800 1.42318800

O 2.45211300 0.88969600 2.17875300

Rh 0.31564500 0.00543500 0.08968000

**1f-TS1**

E = -415.1771493 a.u.

C 2.06997800 0.08419200 -1.95301700

C 0.70731800 0.08020700 -1.85718900

H 2.50693800 0.06137300 -2.95049200

H 0.15179300 0.04425600 -2.79609100

C 2.79535400 0.32097200 0.48465900

C 3.00557600 0.13699900 -0.88031300

H 3.68969900 0.39458400 1.10320900

H 4.05130600 0.10981200 -1.18721400

P 0.50162700 -2.20077100 0.26425900

H 1.31121500 -2.36013500 1.41934800

H -0.50912200 -3.17172900 0.50463500

H 1.28596900 -2.85455500 -0.73051700

P -1.17936900 2.25129700 -0.27525200

H -2.42741600 2.45542600 -0.93117000

H -1.44064900 2.82369100 1.00030100

H -0.39468900 3.26595800 -0.89247400

P 1.30766600 0.90812800 1.21469300

Cl -0.95277300 -0.28610500 2.27050000

C -2.08461400 -0.79806400 -1.05828200

O -3.05345800 -1.19732000 -1.50532000

Rh -0.35579500 0.00773400 -0.16813600

**1f-IN1**

E = -415.201634572 a.u.

C -0.67635200 -1.02804600 2.38838000

C 0.43729400 -0.67806300 1.72471600

H -0.51448900 -1.43786700 3.38687100

H 1.41431500 -0.73547600 2.20590400

C -2.52253900 -0.26059200 0.89517300

C -2.05750400 -0.99999700 1.92860600

H -3.57666500 -0.29196600 0.62744800

H -2.76211700 -1.61471000 2.48504500

P -0.42428900 -2.01975500 -0.89347800

H -1.66740200 -1.93873600 -1.57672400

H 0.31768500 -2.81676300 -1.81458100

H -0.70439600 -2.98358000 0.11327900

P 1.44677800 2.11192100 0.71286500

H 1.19467500 3.25782700 -0.09549800

H 0.88563700 2.53762600 1.94983900

H 2.83800600 2.28875300 0.97708500

Cl -1.97186800 0.96853600 -1.88729000

P -1.51150700 1.06540800 0.17125500

Rh 0.62088900 0.02185600 -0.17260300

C 2.57120800 -0.70717300 -0.63421700

O 3.62239400 -1.12926300 -0.76040100

**2f**

E = -415.2245711 a.u.

C 1.60104100 -2.40703100 0.05413300

C 0.30135000 -2.08599300 0.30587700

H 1.90599200 -3.44709500 0.16870200

H -0.36688000 -2.93554500 0.47582700

C 2.46280700 -0.20423300 -0.75097100

C 2.64097800 -1.49239000 -0.32856300

H 3.32786700 0.43515600 -0.91188400

H 3.66280500 -1.86268800 -0.26374100

P 0.82873400 0.30377500 2.12060500

H 1.39202700 1.61211000 2.11573100

H 0.25192000 0.28128000 3.42447500

H 1.97900100 -0.50687900 2.31657600

P -2.24403700 -1.25309700 -1.21948900

H -3.55078600 -0.70376100 -1.36984700

H -1.83674700 -1.28299900 -2.58367100

H -2.58328400 -2.62604000 -1.03564200

Cl 1.20350100 2.46924400 -1.05483400

P 0.88269200 0.42205000 -1.31598300

C -1.73361000 1.40786100 0.57521700

O -2.36444800 2.35500200 0.67242400

Rh -0.65271300 -0.29291000 0.38455800

**2f-T1**

E_T1_ = -415.185857988 a.u.

C 1.05980200 -2.65619100 -0.01015500

C -0.17776500 -2.09078200 -0.00818300

H 1.09925900 -3.74542100 -0.01445800

H -1.02765900 -2.78275100 -0.01151200

C 2.63790900 -0.66282000 -0.00443000

C 2.34690500 -2.01837300 -0.00781600

H 3.67416900 -0.33605600 -0.00422100

H 3.20536300 -2.68561900 -0.00955300

P -0.93519700 -0.50647800 2.39568500

H -1.25978900 0.56249800 3.28157800

H -1.83098200 -1.50641200 2.87513500

H 0.28239200 -0.95927600 2.97513500

P -0.95336800 -0.48982400 -2.39471100

H -1.31173700 0.57729900 -3.26978200

H 0.26707300 -0.91197200 -2.99128000

H -1.83221800 -1.50623900 -2.87076100

Cl 2.35775900 2.39211000 -0.00251900

P 1.41046600 0.56936700 0.00004100

C -1.81739600 1.64351800 0.01067300

O -2.33181300 2.66257000 0.01407100

Rh -0.88485600 -0.16801200 0.00164900

**1g**

E = -305.3930665 a.u.

C 2.43579700 -0.20319100 1.71859500

C 1.05146900 -0.09262200 1.64378200

H 2.87847400 -0.38497800 2.69606300

H 0.57578300 -0.11991300 2.63047400

C 3.04074100 0.40182500 -0.65220900

C 3.33308300 0.00666500 0.66680500

H 3.90136900 0.60885900 -1.28793400

H 4.38946100 -0.06953900 0.92228600

P -0.96309800 2.24648100 0.35719400

H -0.78055100 3.05857900 -0.79462500

H -2.36419400 2.37357300 0.56484800

H -0.44540600 3.08806200 1.38473100

P 0.42515600 -2.11920500 -0.74854900

H -0.50121200 -3.17600300 -0.51861600

H 0.55048600 -2.17125900 -2.16197300

H 1.64337300 -2.71288400 -0.31054800

P 1.50725300 0.79386400 -1.35659300

P -2.16809100 -0.89035300 1.32097700

H -2.14409400 -1.21728000 2.71299500

H -3.34529800 -0.08969900 1.28627600

H -2.71208500 -2.10290900 0.80836100

Cl -1.81891500 0.02265100 -1.76930600

Ir -0.15466700 0.01808400 0.10764400

**1g-T1**

E_T1_ = -305.352367530 a.u.

C 2.48754800 0.30168200 1.63047300

C 1.12278000 0.31836300 1.53364200

H 2.89913600 0.45892700 2.62681700

H 0.63189600 0.51594400 2.48888100

C 3.21662300 -0.04221400 -0.75377200

C 3.46166400 0.11661100 0.60963800

H 4.05792800 -0.14227000 -1.43538900

H 4.50489000 0.11496500 0.91755200

P -0.42521400 2.37150600 -0.33903300

H 0.45621200 2.96768100 -1.28335500

H -1.69049600 2.81324200 -0.81906900

H -0.22572100 3.22376800 0.78568500

P 0.03453600 -2.35140700 0.10971700

H -0.93334400 -3.09182200 0.85074600

H -0.02564900 -3.01182800 -1.14689200

H 1.24690500 -2.85194000 0.66064600

P 1.63921700 -0.05070800 -1.45128800

P -2.06693600 0.02375700 1.55566600

H -1.78287500 0.25312600 2.93653200

H -3.09570200 0.97857000 1.32665000

H -2.83282100 -1.17225800 1.62811300

Cl -2.14726500 -0.37296000 -1.49629100

Ir -0.20990400 0.01584900 -0.00449100

**1g-TS1**

E = -305.3763331 a.u.

C -1.78474600 -1.14897900 -1.86837600

C -0.44570700 -0.98371500 -1.65434900

H -2.09120600 -1.74094300 -2.72940000

H 0.21827000 -1.45108000 -2.38639000

C -2.88247800 0.31059400 -0.05276700

C -2.86511800 -0.60957800 -1.09001700

H -3.86319300 0.61441700 0.31058400

H -3.85278900 -0.92054300 -1.43234500

P 1.27086900 1.71211000 -1.36382600

H 1.24093100 2.99670700 -0.75035300

H 2.64841500 1.62830500 -1.72602500

H 0.69286500 2.00016600 -2.63326400

P -0.75693700 -1.63600900 1.35225300

H 0.10941600 -2.36763900 2.21669600

H -1.69716600 -1.14230400 2.29369500

H -1.47808900 -2.69935800 0.73851900

P -1.52168500 1.42421400 0.23843000

P 2.40233700 -1.31760200 0.01856200

H 2.52050400 -2.50073300 -0.77333500

H 3.61558200 -0.67573800 -0.37252400

H 2.83694200 -1.84521200 1.27066300

Cl 0.16985300 1.29002200 2.16170400

Ir 0.30079900 -0.02567600 -0.08394400

**1g-IN1**

E = -305.3864452 a.u.

C 0.77477000 -0.58407300 2.58165400

C -0.33552400 -0.33357100 1.86114200

H 0.60995900 -0.82934300 3.63115600

H -1.30197300 -0.32998300 2.37061100

C 2.66217200 -0.08876000 0.99554000

C 2.16166100 -0.61385700 2.13958400

H 3.73278000 -0.14013200 0.80816600

H 2.86368900 -1.07859700 2.82976600

P -1.23532300 2.24240500 0.36748900

H -1.07894300 3.19214800 -0.68345500

H -2.59934200 2.47771000 0.71913200

H -0.56759800 2.91652300 1.42883300

P 0.52233500 -2.09683600 -0.50145900

H -0.07104100 -2.92415100 -1.50191300

H 1.88573500 -2.16521300 -0.88898400

H 0.48557300 -2.97963500 0.61435500

P 1.69784400 1.04752400 -0.05236300

P -2.77116300 -0.88661900 -0.48363000

H -3.15238900 -1.99901200 0.32595700

H -3.93029500 -0.07702700 -0.27999900

H -3.12260000 -1.42739000 -1.75741800

Cl 2.20967500 0.44530600 -2.03600900

Ir -0.46885600 0.03790100 -0.10937800

**2g**

E = -305.4093461 a.u.

C 1.77111800 -2.36663800 0.07509900

C 0.45476700 -2.08797700 0.29521800

H 2.11925100 -3.38854800 0.21872400

H -0.17518500 -2.96322800 0.49041200

C 2.57286700 -0.14531300 -0.78644800

C 2.77390800 -1.42143900 -0.34098300

H 3.43533200 0.48115700 -1.00547800

H 3.80535900 -1.76757800 -0.29118300

P 0.93011800 0.38075600 2.02370900

H 1.53182500 1.66913700 1.94560000

H 0.33208500 0.45721600 3.31717800

H 2.04631300 -0.45527600 2.29868900

P -1.97524400 -1.27641100 -1.35110700

H -3.07816100 -0.53733900 -1.87342500

H -1.36985100 -1.70518200 -2.56610000

H -2.64966800 -2.47437100 -0.97030500

Cl 1.30428700 2.55607700 -1.00596900

P 0.98270400 0.48630800 -1.34856000

P -1.83560700 1.75380000 0.44020000

H -2.22006700 2.40842400 -0.76738500

H -3.12013800 1.66704600 1.05725300

H -1.33393100 2.88618900 1.14656600

Ir -0.49950100 -0.30869600 0.28805200

**2g-T1**

E_T1_ = -305.366362006 a.u.

C 1.45324900 -2.57349000 0.00156300

C 0.16418600 -2.12023500 0.00200200

H 1.59776700 -3.65308600 0.00272500

H -0.59559400 -2.91045600 0.00381600

C 2.84205600 -0.43850500 -0.00121000

C 2.67123200 -1.81729900 0.00005800

H 3.84457400 -0.02113100 -0.00186100

H 3.58910000 -2.40100400 0.00017100

P -0.72477400 -0.52267400 2.36614400

H -1.07004000 0.56184200 3.22711700

H -1.60052900 -1.52671200 2.87435200

H 0.50302200 -0.93698600 2.95446500

P -0.72756700 -0.52690200 -2.36469200

H -1.06768200 0.55842600 -3.22668700

H 0.49766100 -0.94840400 -2.95322700

H -1.60893400 -1.52692600 -2.87111200

Cl 2.29259100 2.59375600 -0.00143600

P 1.51021700 0.67551300 -0.00223600

P -1.93253500 1.84480400 -0.00053700

H -1.76715400 2.75056200 -1.09071100

H -3.35871500 1.79398300 0.01790200

H -1.73980700 2.76651800 1.07154800

Ir -0.66054800 -0.24629100 0.00027600

**1h**

E = -410.3765178 a.u.

C 2.47649600 -0.00845500 1.67225000

C 1.08893900 -0.00820800 1.64431800

H 2.95059000 -0.02131400 2.65134000

H 0.66788400 -0.01705900 2.65266700

C 3.01363500 0.03739800 -0.79193000

C 3.34305500 0.01068500 0.57632500

H 3.85791200 0.05519200 -1.48072600

H 4.40608800 0.00886900 0.81535700

P -0.41595700 2.35198000 -0.18848300

H -0.15748900 2.76432200 -1.52208700

H -1.72482500 2.85736000 0.03205200

H 0.38978400 3.24614600 0.57259700

P -0.32000400 -2.34823100 -0.27141800

H -1.60428600 -2.91635800 -0.05919500

H -0.05657500 -2.70316200 -1.62027600

H 0.53009600 -3.23269300 0.45205500

P 1.46396300 0.05547100 -1.55472100

Cl -1.83029400 0.00130600 -1.74508800

C -1.74494300 -0.05311600 1.44933100

O -2.61763400 -0.08236700 2.18667400

Ir -0.22319800 -0.00211300 0.16359600

**1h-T1**

E_T1_ = -410.336360896 a.u.

C -2.48210600 -0.26908500 1.60955100

C -1.11247000 -0.24293400 1.59067000

H -2.94132400 -0.32821700 2.59562100

H -0.67270100 -0.34649000 2.58336000

C -3.10762400 -0.34052300 -0.82532100

C -3.41031600 -0.22453100 0.52944100

H -3.92125300 -0.30850400 -1.54741600

H -4.46030400 -0.10885300 0.78744700

P 1.12168900 -2.23492900 0.16583200

H 1.01446700 -2.92768800 -1.06862700

H 2.50891600 -2.34906100 0.45176300

H 0.54407000 -3.15520600 1.08631700

P -0.43094400 2.25388400 -0.39098600

H 0.35250300 3.29882200 0.17395900

H -0.40086300 2.57899700 -1.77238300

H -1.74579300 2.62272600 0.00533000

P -1.51862500 -0.68242900 -1.42098300

Cl 1.96671800 0.25146300 -1.69889800

C 1.54034800 0.60545800 1.38454300

O 2.27716000 0.97084100 2.18180000

Ir 0.28470200 0.02290500 0.09208100

**1h-TS1**

E = -410.3661712 a.u.

C 1.91212900 0.68994200 -1.96684900

C 0.56183500 0.70217500 -1.76722800

H 2.28239600 1.10489500 -2.90281600

H -0.03251100 1.14021400 -2.56989700

C 2.84469300 -0.53232100 0.09686100

C 2.92631600 0.19394300 -1.08541400

H 3.79138900 -0.84131200 0.53807300

H 3.94225400 0.34786700 -1.45082400

P -1.37268900 -1.82791500 -1.09232200

H -1.41103300 -2.97922200 -0.25808600

H -2.73597400 -1.71684900 -1.48772400

H -0.78260900 -2.34007500 -2.28125900

P 0.78103200 1.82351200 1.12668300

H -0.08461300 2.73862200 1.78749900

H 1.61399300 1.40136300 2.19433300

H 1.62679800 2.70231400 0.39356500

P 1.40143900 -1.45093300 0.55724700

Cl -0.50349900 -0.81855400 2.28245300

C -1.93187100 1.19896800 -0.34321500

O -2.88455200 1.81633100 -0.50051000

Ir -0.34544900 0.07975000 -0.09402000

**1h-IN1**

E = -410.3758677 a.u.

C 0.99751200 -1.25216000 2.22273200

C -0.19628100 -0.93200800 1.69140800

H 0.96066700 -1.79229000 3.16907400

H -1.10806600 -1.18405900 2.23183200

C 2.70925200 -0.15661800 0.71960900

C 2.33632900 -1.04263300 1.67666300

H 3.76130200 -0.07241700 0.45530900

H 3.12000200 -1.64978600 2.12709000

P -1.44683100 1.87950200 1.00445700

H -1.21076900 3.13572800 0.37872000

H -2.85742300 1.92664500 1.19943400

H -0.96834700 2.12312300 2.32195600

P 0.68240100 -1.85573600 -1.11452100

H -0.00757100 -2.50197900 -2.18027900

H 1.94224500 -1.60739400 -1.71729800

H 0.95786100 -2.95506200 -0.25671500

P 1.58572900 1.20623400 0.27139600

Cl 1.47960800 1.31523200 -1.87839400

C -2.22845500 -0.81235700 -0.51106100

O -3.26203900 -1.28115200 -0.67919800

Ir -0.48930600 -0.01048800 -0.09842700

**2h**

E = -410.4026578 a.u.

C 1.31404800 -2.60960400 -0.02831500

C 0.06140400 -2.10839900 0.18196500

H 1.47095200 -3.68425100 0.05554700

H -0.71398000 -2.87394400 0.29013900

C 2.50787100 -0.52383900 -0.74257900

C 2.48001600 -1.83588900 -0.35594400

H 3.46860400 -0.02964600 -0.87342500

H 3.43737900 -2.34983100 -0.28227000

P 0.94164600 0.17920500 2.06665300

H 1.77664400 1.32951200 2.00957100

H 0.33887900 0.34632100 3.34671100

H 1.86623000 -0.86743600 2.32519500

P -2.24301200 -0.79667400 -1.36155400

H -3.44216300 -0.03503200 -1.46167100

H -1.81761700 -0.79866700 -2.71942800

H -2.77656400 -2.11234600 -1.24719800

Cl 1.72862300 2.33589200 -0.97860200

P 1.07424300 0.37984600 -1.34492300

C -1.35711600 1.61343100 0.50171400

O -1.84234100 2.64640600 0.61672100

Ir -0.58294500 -0.17475200 0.29241100

**2h-T1**

E_T1_ = -410.360033537 a.u.

C -1.08460400 -2.70930200 0.00096000

C 0.12664300 -2.07826800 0.00165300

H -1.07893900 -3.79833700 0.00192800

H 0.98523300 -2.75974500 0.00336600

C -2.75338700 -0.78779500 -0.00173900

C -2.39669800 -2.13011500 -0.00042800

H -3.80346500 -0.50847600 -0.00224300

H -3.22604100 -2.83339600 -0.00014100

P 0.85243200 -0.39506200 -2.37646900

H 1.19523600 0.70212800 -3.21768800

H 1.78373100 -1.37513200 -2.82462600

H -0.34205700 -0.85606700 -2.99549700

P 0.84596800 -0.39131100 2.37870600

H 1.18797900 0.70663200 3.21927500

H -0.35080800 -0.84984800 2.99512300

H 1.77464300 -1.37199900 2.83090900

Cl -2.60032700 2.28850100 -0.00235100

P -1.59219200 0.50114900 -0.00352900

C 1.52293300 1.69372600 0.00021600

O 1.98959100 2.74133500 0.00010700

Ir 0.72832300 -0.09794900 0.00057300

**1i**

E = -362.8611226 a.u.

C -2.75501000 0.13271000 1.08736700

C -1.33227300 -0.21447200 1.50635500

H -3.42422000 -0.09935000 1.92722000

H -0.98207100 0.44329000 2.30268200

C -2.39102300 -0.31709500 -1.38894700

C -3.26588400 -0.59949400 -0.14326700

H -2.83186700 -0.82496100 -2.25526200

H -3.28269100 -1.68027300 0.03286600

Cl 1.63498500 0.72015800 1.78011600

Cl 2.04357200 0.32689900 -1.57905300

P 1.09267200 -2.21589000 0.24894900

H 1.05392900 -3.07724600 -0.88347500

H 2.47259300 -2.20121800 0.57819400

H 0.57779900 -3.08702100 1.25767600

P -0.27524700 2.26119700 -0.27472300

H 0.94033700 2.98800400 -0.35775600

H -0.98956500 2.80911400 -1.38634300

H -0.93386000 2.93428500 0.79620500

H -1.29519600 -1.24063400 1.88685700

H -2.85160300 1.21379700 0.92183100

Ir 0.12598000 -0.06388400 -0.05760100

H -4.28918200 -0.29222700 -0.38390900

H -2.38893500 0.76765600 -1.57805400

N -1.11538200 -0.88908300 -1.11771100

**2i**

E = -362.8477055 a.u.

C 1.30817000 -1.42625700 1.98372100

C -0.01314000 -0.69145000 1.84189800

H 1.40343100 -1.73813700 3.03413700

H -0.86216800 -1.34553500 2.07005300

C 2.64436500 -0.28783400 0.11360800

C 2.54070500 -0.61238600 1.60042400

H 3.61867200 0.17618900 -0.08655700

H 2.56146900 0.32335300 2.17265100

Cl -2.79405900 -0.27517100 -0.11944800

Cl 1.71006800 0.95888300 -2.01313000

P -0.58636000 2.24715200 0.80794900

H 0.20604100 2.58167000 1.94734100

H -0.20121900 3.31034700 -0.06159900

H -1.86438700 2.71600000 1.23415800

P -0.35028200 -2.07396600 -1.00981900

H -1.10314900 -2.19561100 -2.21331100

H 0.83951700 -2.75740700 -1.41664300

H -0.96280300 -3.11279100 -0.24751100

H -0.04094000 0.13380400 2.56348600

H 1.29729200 -2.35878600 1.40210400

Ir -0.37061700 0.07716200 -0.05244300

H 3.44244200 -1.16923300 1.87977000

H 2.61187400 -1.22451600 -0.46810300

N 1.59833100 0.65797500 -0.24684100

**1j**

E = -314.6861879 a.u.

C 2.62202400 0.81071500 -1.16474800

C 1.19005900 0.47240000 -1.55921300

H 3.16950000 1.07291800 -2.08115300

H 0.72655400 1.32976400 -2.05337800

C 2.89591600 -0.63896000 0.94667300

C 3.40557000 -0.29016600 -0.46332400

H 3.58211500 -1.34027400 1.43238500

H 3.41034600 -1.19270400 -1.08945900

Cl -1.79823800 1.55353000 -1.15947200

Cl -2.04133500 -0.57208500 1.63194700

P -1.33936100 -1.73938400 -1.17631300

H -1.40575900 -3.02795900 -0.57473400

H -2.70726000 -1.42007100 -1.36577300

H -0.94268100 -2.09256900 -2.50451200

P 0.23519400 1.98122800 1.13759300

H -0.91541600 2.45021100 1.82358500

H 1.21570500 2.07643000 2.17717400

H 0.59358200 3.11355900 0.35330400

H 1.20914500 -0.34391200 -2.29178500

H 2.64768600 1.71559400 -0.54171100

Ir -0.19486800 -0.04121700 -0.01835600

H 4.45584600 0.01045000 -0.36870500

H 2.85529300 0.26385900 1.56661100

P 1.21760000 -1.45851700 0.86160000

**2j**

E = -314.7177628 a.u.

C -2.12103600 -1.61133100 1.36137600

C -0.68331200 -1.79531200 0.90062800

H -2.44457200 -2.54040700 1.85174100

H -0.07504700 -2.11605300 1.76075100

C -2.87621600 0.07413900 -0.38958100

C -3.10476100 -1.29487900 0.24130600

H -3.63543400 0.27906300 -1.15290100

H -3.04139000 -2.07775700 -0.52660400

Cl 2.72101400 0.86413200 0.31614900

Cl -1.19750700 2.33668300 -1.40538000

P 1.50275400 -1.58034500 -1.44833500

H 0.78673900 -2.70373700 -1.96916200

H 1.86592500 -0.94177300 -2.66911700

H 2.74435000 -2.20598900 -1.12938800

P -0.15095800 1.37523100 1.78802600

H 0.46618200 2.65175500 1.67152100

H -1.50940800 1.77112900 1.97453200

H 0.17559400 1.08136200 3.14847900

H -0.66999200 -2.62932400 0.18595300

H -2.18575400 -0.83286200 2.13396100

Ir 0.51404700 -0.20246000 0.17920600

H -4.13019200 -1.33483200 0.62766700

H -2.96210500 0.86618800 0.35937500

P -1.22874300 0.18331900 -1.28843000

**1k**

E = -347.4455909 a.u.

C -0.25420200 -0.73690000 0.00006900

C -0.25440800 0.73691500 0.00001900

C 2.15958600 -0.72394300 -0.00027600

C 1.00454000 -1.43712500 -0.00021900

H 3.11598500 -1.23909300 -0.00046000

H 1.01583500 -2.52347500 -0.00040700

C -1.54031300 1.18025500 -0.00005800

C -2.45146100 -0.00016500 0.00018200

C -1.54000700 -1.18040400 0.00021500

C 1.00420600 1.43724300 -0.00014000

H 1.01539300 2.52357700 -0.00060200

H -3.11853000 -0.00025000 -0.87430900

H -3.11829200 -0.00005100 0.87486000

H -1.87530700 2.21122100 -0.00019600

H -1.87477100 -2.21141800 0.00024800

C 2.15939800 0.72412700 0.00045200

H 3.11565200 1.23948100 -0.00060000

**2k**

E = -347.4818619 a.u.

C 0.23233200 -0.68656800 0.00003800

C 0.21302300 0.72109300 -0.00005800

C -2.16500400 -0.71345900 -0.00021800

C -0.95204700 -1.40798300 -0.00013700

H -3.10091700 -1.26483600 -0.00028600

H -0.94343300 -2.49516000 -0.00032000

C 1.59484500 1.19377400 -0.00010500

C 2.43163100 0.13954300 -0.00004300

C 1.66161700 -1.14857300 0.00002100

H 1.89759800 -1.76467000 0.87773300

H 1.89758100 -1.76481800 -0.87759200

H 3.51501900 0.18415000 -0.00006300

H 1.88245700 2.23999900 -0.00019400

C -0.99696900 1.41282700 -0.00017800

H -1.01672600 2.49939800 -0.00011700

C -2.18513200 0.68272300 0.00078100

H -3.13735100 1.20567400 0.00022400

**1l**

E = -315.2477881 a.u.

C 0.63687000 0.75912300 0.00006300

C 0.49262300 -0.70902400 0.00000800

C -1.78106400 1.19142900 -0.00013000

C -0.50346600 1.64054200 -0.00000400

H -2.58952900 1.91907500 -0.00016900

H -0.30512200 2.71176100 0.00005600

C 1.73787500 -1.26766000 0.00009400

C 2.75769100 -0.18353000 0.00025500

C 1.95994300 1.07407300 0.00018800

C -0.78927100 -1.35280700 -0.00011200

H -0.79961700 -2.44144500 -0.00013400

H 3.42214600 -0.24480900 -0.87429500

H 3.42160200 -0.24483300 0.87523500

H 1.96872600 -2.32649100 0.00009100

H 2.38672900 2.07023800 0.00025500

P -2.30481000 -0.55709100 -0.00021400

**2l**

E = -315.2830301 a.u.

C 0.60864000 0.71035100 0.00002300

C 0.46152000 -0.69357500 -0.00000300

C -1.78862000 1.15271100 -0.00001100

C -0.46525300 1.58929000 0.00001900

H -2.57239300 1.90698500 -0.00001100

H -0.26371300 2.66017400 0.00004100

C 1.80251600 -1.28134700 0.00001000

C 2.72816300 -0.30545500 0.00004000

C 2.07333800 1.04197100 0.00005300

H 2.36021400 1.63645500 -0.87785900

H 2.36018100 1.63641600 0.87800200

H 3.80342500 -0.44442000 0.00005500

H 1.99253700 -2.34924500 -0.00000300

C -0.76839000 -1.34353400 -0.00003400

H -0.78225200 -2.43196600 -0.00005200

P -2.32063300 -0.52245800 -0.00005000

**1m**

E = -427.6842402 a.u.

C -2.70999100 0.39679700 -0.00004100

C -1.53468100 -0.50659200 -0.00020700

C -1.64837200 2.66596000 0.00024400

C -2.73116500 1.82621400 0.00020800

H -1.85528300 3.73611000 0.00037900

H -3.72539200 2.27634100 0.00031800

Cl 1.17922300 -2.37724800 0.00036800

Cl 2.86853800 0.72504300 0.00014700

P 0.64789100 -0.30297600 -2.31992100

H 0.49626900 0.76110900 -3.26192400

H 1.92126200 -0.81141600 -2.68448500

H -0.23973000 -1.26747300 -2.87502200

P 0.64701300 -0.30264600 2.32002900

H 1.92033000 -0.81077700 2.68522500

H 0.49475700 0.76150100 3.26186600

H -0.24064600 -1.26727400 2.87483000

C -2.03233500 -1.77085700 -0.00049400

C -3.52906300 -1.77453800 -0.00047500

C -3.86720900 -0.33325500 -0.00018000

H -3.95848900 -2.28574700 0.87380800

H -3.95852000 -2.28539200 -0.87495000

H -1.44000000 -2.67769600 -0.00065400

H -4.87532800 0.06901400 -0.00008900

Ir 0.44393900 0.05411100 -0.00000600

P 0.09058900 2.25230900 -0.00023500

**2m**

E = -427.7088576 a.u.

C -2.63801100 0.36263100 -0.02786800

C -1.49530600 -0.46832400 -0.16842600

C -1.58011400 2.62626600 -0.18283100

C -2.66659100 1.74922400 -0.01673400

H -1.82955500 3.68596300 -0.23825300

H -3.65587100 2.20630700 0.05294800

Cl 1.01867600 -2.34289600 0.72554500

Cl 2.84515200 0.64341300 0.14374200

P 1.15288000 -0.60677000 -2.17516200

H 1.74305200 0.39556200 -2.99509600

H 2.17851700 -1.58587100 -2.12008500

H 0.24876900 -1.20329100 -3.10900200

P 0.28513400 0.26457100 2.30812400

H -0.65757100 -0.55991300 2.98406500

H 1.50039400 -0.07662600 2.95471500

H -0.00870800 1.53340300 2.89573100

C -1.99834800 -1.82307800 -0.36268500

C -3.34969200 -1.84536200 -0.29590800

C -3.88675500 -0.48040500 -0.06024900

H -4.44785700 -0.42756200 0.88315500

H -4.58573100 -0.17246100 -0.84906200

H -3.97503100 -2.72785700 -0.38463100

H -1.35214400 -2.68078700 -0.49239200

Ir 0.41458700 0.01656700 -0.03169600

P 0.09014600 2.24239800 -0.48170800

**1n**

E = -295.8205859 a.u.

C -1.85049500 -0.62677200 1.98647800

C -0.51806900 -0.51043700 1.61806400

H -2.09682800 -1.03804900 2.96498900

H 0.14618400 -0.89601200 2.39827000

C -2.92157600 0.43902600 -0.03214100

C -2.94274400 -0.20835900 1.21634700

H -3.89122100 0.74550000 -0.42549700

H -3.92716800 -0.36146200 1.65816100

P 2.56907300 -0.91547700 0.64177700

H 3.76601300 -0.14255100 0.54328900

H 2.71642600 -1.33976300 1.99933200

H 3.06486900 -2.09916800 0.01342100

P -1.58298600 0.96869800 -0.98946900

P 1.18562100 2.26002600 -0.02154300

H 0.96111800 3.05560400 1.14205100

H 2.59294200 2.43019300 -0.16017300

H 0.72080900 3.16079900 -1.02083800

P -0.28554500 -1.87102100 -1.31507800

H -0.71953700 -1.71236800 -2.66036900

H 0.69823600 -2.88662100 -1.49625800

H -1.35924100 -2.64929500 -0.79310800

Rh 0.38396000 0.04830000 -0.13388300

H 1.15405700 0.36553400 -1.60634100

**1n-TS1**

E = -295.8075707 a.u.

C -1.86107600 -0.59270900 1.80316200

C -0.51334700 -0.59660300 1.57024900

H -2.20755100 -0.96791600 2.76736200

H 0.09156100 -1.16521400 2.28177800

C -2.82406200 0.44763500 -0.32182300

C -2.89890200 -0.15090800 0.92674600

H -3.77451400 0.71343000 -0.78412200

H -3.90764700 -0.28381500 1.31756000

P 2.60326700 -1.00736900 0.21545200

H 3.74504100 -0.15911000 0.34491700

H 2.82299600 -1.79903100 1.38694900

H 3.16297900 -1.91921100 -0.73132900

P -1.40719100 1.27065200 -0.99194100

P 1.24577400 2.23583700 0.28892600

H 0.89508800 2.85476600 1.52344800

H 2.66387600 2.38843400 0.33994600

H 0.91178800 3.28472500 -0.61589700

P -0.47258800 -2.03664500 -0.80369700

H -1.87825200 -2.16881500 -0.97845500

H 0.01123400 -2.54049200 -2.04532000

H -0.19901800 -3.15371200 0.03728000

Rh 0.36663500 0.06488900 -0.16576800

H 0.20923900 0.41435300 -1.88564300

**1n-IN1**

E = -295.8575135 a.u.

C -1.59927200 -0.01411300 2.00210400

C -0.32466800 -0.00557700 1.57949500

H -1.71046500 -0.02787400 3.08817600

H 0.49521300 0.06599700 2.29760900

C -2.96658800 0.09022300 -0.08814500

C -2.83849000 -0.05558200 1.24855700

H -3.96065900 0.00602600 -0.52554400

H -3.73943300 -0.23565300 1.83088200

P 2.88166200 -0.58891100 0.33109300

H 3.94235600 0.36854700 0.35275700

H 3.08936600 -1.12616600 1.63869200

H 3.57161100 -1.60610600 -0.39657800

P -1.66523300 0.58655800 -1.25845300

P 0.74519900 2.39068500 -0.05646300

H -0.39893500 3.12200800 0.37218500

H 1.73233700 2.94881600 0.81361600

H 1.05544900 3.10628400 -1.25080600

P -0.27455600 -2.23196200 -0.40848900

H -1.58445200 -2.49169400 0.08073900

H -0.34872000 -2.83562700 -1.69959300

H 0.46154100 -3.24130200 0.28169000

Rh 0.45163300 0.00037200 -0.27260300

H -1.86068100 -0.50524000 -2.18407600

**2n**

E = -295.8723265 a.u.

C 2.47853600 0.98629600 -0.89101100

C 1.15587300 0.95711800 -1.20317500

H 3.14667900 1.51102000 -1.57449800

H 0.84590300 1.59246100 -2.04025800

C 2.45306000 -0.11561200 1.33812300

C 3.10985200 0.37173000 0.24834500

H 3.01585900 -0.65725500 2.09642500

H 4.19382700 0.27731200 0.21456100

P 0.72904400 0.23644900 1.71431600

P -2.45153300 -1.16567800 0.43940100

H -3.32962900 -1.76295300 -0.51588900

H -2.32155200 -2.29350100 1.30744300

H -3.44307100 -0.46578100 1.19305500

P 0.71267400 -2.00056900 -0.77428900

H 0.75894500 -2.99708800 0.24638100

H 0.19192800 -2.79332400 -1.84166600

H 2.08304600 -1.93012600 -1.14431200

P -1.45060800 2.19474000 -0.10193200

H -2.48423200 2.63848100 -0.98364800

H -2.08511500 2.47742900 1.14361000

H -0.58395400 3.32358500 -0.17986400

H 0.47605800 -0.99938900 2.41870800

Rh -0.41649500 -0.00204900 -0.36569300

**1o**

E = -291.0125291 a.u.

C -1.88721700 -0.49218900 2.00489900

C -0.54682500 -0.37923600 1.66157900

H -2.14637400 -0.83663700 3.00508100

H 0.10015600 -0.67082500 2.49595600

C -2.95545200 0.49738700 -0.06634600

C -2.97243700 -0.11427700 1.19869800

H -3.92680300 0.80183000 -0.45571700

H -3.95781500 -0.24192600 1.64742200

P 2.42432700 -1.05274200 0.56745200

H 3.64943900 -0.33324100 0.43480500

H 2.56884300 -1.48652700 1.92165900

H 2.82395200 -2.25567100 -0.08875400

P -1.59891200 1.07561500 -0.98577100

P 1.27562400 2.18154900 0.06746300

H 1.09466500 2.93854500 1.26228300

H 2.68986300 2.27623000 -0.07627000

H 0.85044000 3.13344600 -0.90169400

P -0.55760200 -1.91779500 -1.10257600

H -1.01890900 -1.81039400 -2.44264700

H 0.31028600 -3.04228100 -1.22437700

H -1.68410800 -2.51858500 -0.47112200

Ir 0.31969000 0.03251000 -0.13540500

H 1.05027500 0.17329000 -1.67193400

**1o-TS1**

E = -290.9892209 a.u.

C -1.75217900 -0.23262300 1.97552300

C -0.40995600 -0.28031100 1.71764600

H -2.07169400 -0.41491900 3.00139200

H 0.22536700 -0.61410700 2.54158300

C -2.83055500 0.41815400 -0.27663400

C -2.82340500 0.01468100 1.05047900

H -3.81153700 0.57265900 -0.72530800

H -3.81594000 -0.10291700 1.48763500

P 2.36368200 -1.22590900 0.23380700

H 3.59992400 -0.52869900 0.08139300

H 2.60608500 -1.80384200 1.51998800

H 2.67426400 -2.36802700 -0.56569200

P -1.44910100 1.29232200 -1.00364800

P 1.40836400 2.10151000 0.19456000

H 1.15724300 2.77172100 1.42568000

H 2.83456600 2.13715000 0.17459900

H 1.10805000 3.14421100 -0.72948100

P -0.81825300 -1.99057900 -0.71480100

H -1.74201500 -1.93488100 -1.79325700

H 0.01529000 -3.06114800 -1.15491500

H -1.58229000 -2.65539400 0.28621000

Ir 0.30221200 0.03083200 -0.14374400

H -0.14143600 0.30455700 -1.93234000

**1o-IN1**

E = -291.0266426 a.u.

C 1.65543000 -0.27298700 2.02797800

C 0.36847900 -0.18739400 1.64014900

H 1.79281500 -0.44120800 3.09735900

H -0.40557700 -0.33449200 2.39674400

C 3.04346000 0.14561900 -0.03809500

C 2.88742300 -0.17483900 1.26465600

H 4.05628600 0.20019900 -0.43343100

H 3.79508300 -0.36501300 1.83535900

P -2.68478300 -0.51817500 0.41824300

H -3.43798300 -1.49360100 -0.30330900

H -2.88249700 -1.03747000 1.73505800

H -3.67172300 0.51337300 0.45069700

P 1.72774300 0.48629700 -1.25998500

P 0.23639000 -2.24998000 -0.49135500

H 1.63073600 -2.53205700 -0.46080400

H -0.24889100 -3.19434700 0.45997300

H -0.14314000 -2.88941600 -1.70754400

P -0.59953800 2.36186900 0.09616200

H -0.34107600 3.20530700 -1.02389600

H -1.83529900 2.92119400 0.54536800

H 0.29447600 2.91216000 1.05623800

Ir -0.36899600 0.03055200 -0.22302300

H 1.88351600 1.92031800 -1.28911500

**2o**

E = -291.0438772 a.u.

C 2.50977400 1.08261300 -0.80073600

C 1.18046200 1.07501800 -1.10677500

H 3.18208400 1.65496700 -1.43916900

H 0.89238400 1.76643600 -1.90708200

C 2.49380600 -0.18829700 1.35375400

C 3.13401300 0.39246800 0.29776800

H 3.08597800 -0.75956800 2.06728100

H 4.21990200 0.31769000 0.26376800

P 0.77105200 0.09255400 1.81055100

P -2.28612200 -1.23015200 0.49373500

H -2.91946700 -2.16470600 -0.38110100

H -2.13457800 -2.08545000 1.62886400

H -3.45616100 -0.53021000 0.91848200

P 0.80633600 -1.94344700 -0.82344500

H 0.99701800 -2.92615100 0.19304400

H 0.21954000 -2.75937800 -1.83723800

H 2.12586900 -1.79305800 -1.33052200

P -1.39146400 2.16320700 0.05284000

H -1.52023200 3.01055600 -1.08807800

H -2.72037800 2.25192800 0.56505100

H -0.72046200 3.04470000 0.94637000

Ir -0.34058100 0.02304800 -0.29196600

H 0.56786400 -1.20569400 2.41240700

**1p**

E = -335.0923506 a.u.

C -2.12386900 -0.44378500 1.92700700

C -0.75959800 -0.36865200 1.68152000

H -2.47378400 -0.75243900 2.91166200

H -0.18133900 -0.66283700 2.56429200

C -2.97522900 0.40488400 -0.29294000

C -3.12712100 -0.10114300 1.01173700

H -3.89832600 0.66574000 -0.81105500

H -4.15207300 -0.19857300 1.36871500

P 2.42870000 -0.83262700 1.03558700

H 3.59591900 -0.00957600 1.00284100

H 2.47857300 -1.19466100 2.41839400

H 3.02803900 -2.01610500 0.50384800

P -1.54060800 0.81041500 -1.16783200

P 0.95694300 2.27736800 0.22197700

H 0.41456900 3.08753100 1.26278100

H 2.34672600 2.53274100 0.41553800

H 0.71183000 3.12100400 -0.89987100

P -0.24551000 -2.03022900 -1.02223100

H -0.51483100 -2.01115100 -2.42029400

H 0.74962500 -3.05225200 -0.98772800

H -1.37487200 -2.76175400 -0.55237600

Rh 0.32279600 0.01546900 -0.02126600

C 1.55584600 0.23623300 -1.86629500

H 2.40172900 0.91858300 -1.72904900

H 1.98551100 -0.72109200 -2.18149000

H 0.94380900 0.61962200 -2.68793700

**1p-TS1**

E = -335.078243 a.u.

C 1.81491700 -0.33958400 -2.01798000

C 0.46774700 -0.33208700 -1.79696600

H 2.17267100 -0.56359500 -3.02336900

H -0.15910400 -0.65731500 -2.63156600

C 2.78765500 0.35664500 0.25633200

C 2.84968000 -0.07881200 -1.06051200

H 3.74474200 0.52630100 0.74975200

H 3.86076800 -0.19858100 -1.45141500

P -2.49800600 -1.16775300 -0.44051200

H -3.69136200 -0.40396100 -0.62938300

H -2.61394500 -1.98018900 -1.61191700

H -3.03540300 -2.10960000 0.49114400

P 1.38284800 1.20353300 0.94531600

P -1.27228800 2.15271200 -0.48097300

H -0.85461900 2.79707700 -1.68081300

H -2.68789200 2.25733300 -0.63776400

H -1.05563900 3.21095100 0.45040700

P 0.66811800 -2.02317400 0.69502200

H 1.43878600 -2.02320600 1.89144200

H -0.22435100 -3.09391200 1.00583500

H 1.55271200 -2.70102500 -0.19063000

Rh -0.33973600 0.03842000 0.04263400

C -0.50469100 0.34216400 2.35318900

H -0.86418800 1.34131400 2.60387800

H -1.30261000 -0.39069900 2.51294700

H 0.30561000 0.09048700 3.04575100

**1p-IN1**

E = -335.1439172 a.u.

C -1.27219300 0.04599900 2.31972500

C -0.05568600 0.04988800 1.74620000

H -1.26233700 0.04670400 3.41123300

H 0.84032000 0.14712300 2.36460000

C -2.84260600 0.19474800 0.39099900

C -2.58442000 -0.00095700 1.70393200

H -3.87169800 0.10662700 0.04431000

H -3.41719900 -0.21490800 2.37037400

P 3.03666400 -0.68678600 -0.03612300

H 3.87329800 0.00337200 0.89482200

H 3.43333200 -2.01314100 0.31915200

H 3.88841900 -0.54698700 -1.17514800

P -1.65866100 0.78055800 -0.85019200

P 0.98459500 2.36479800 -0.04764500

H 0.03242600 3.12379800 0.69032800

H 2.19705400 2.88152600 0.51102200

H 0.97042000 3.08935500 -1.27568900

P -0.26449900 -2.21814700 -0.07438000

H -0.31331300 -3.01546300 -1.25853000

H 0.45628700 -3.11127400 0.77109100

H -1.58607600 -2.40426800 0.41837000

Rh 0.52062000 -0.00663100 -0.18127700

C -2.14044600 -0.28760900 -2.31297600

H -1.38803800 -0.19344200 -3.10017600

H -2.30475400 -1.34206600 -2.07967900

H -3.07544600 0.12269400 -2.71078400

**2p**

E = -335.1608001 a.u.

C 2.38518700 1.45187500 -0.66368200

C 1.04915800 1.49165900 -0.91694600

H 3.01228000 2.21626300 -1.12352300

H 0.69102500 2.37983700 -1.44898600

C 2.48110500 -0.38483300 1.01563100

C 3.07507900 0.48419300 0.14674000

H 3.09819500 -1.13745400 1.50444600

H 4.15834300 0.44619500 0.04367500

P 0.78050400 -0.24405800 1.57434600

P -2.43416100 -1.21506600 -0.09195100

H -3.47949200 -1.15821100 -1.06416900

H -2.31559000 -2.63820900 -0.03484200

H -3.25405200 -1.05677500 1.06839400

P 0.70170800 -1.38681100 -1.67098700

H 0.75717100 -2.73867000 -1.21433700

H 0.19898700 -1.62522200 -2.98610700

H 2.07338700 -1.15364400 -1.96172700

P -1.52758700 2.12632200 0.64958200

H -2.56488900 2.86565900 0.00055800

H -2.16873300 1.91292900 1.90580800

H -0.67275300 3.20724600 1.01281100

Rh -0.45204800 0.22385200 -0.43699800

C 0.46974200 -2.00130000 2.12379500

H 0.68780800 -2.75483400 1.36416500

H 1.11570000 -2.18948500 2.98874600

H -0.56380400 -2.10433300 2.46193900

**1q**

E = -330.2849351 a.u.

C -2.15556500 -0.47650800 1.88772700

C -0.78530900 -0.37597100 1.67060100

H -2.51122600 -0.81379600 2.86023500

H -0.22591300 -0.65406300 2.57128000

C -3.00501900 0.51049800 -0.28030000

C -3.15382600 -0.08780900 0.98370900

H -3.92866600 0.82200500 -0.76737300

H -4.17859400 -0.19631300 1.33865800

P 2.29360600 -1.04190300 0.91064800

H 3.52449200 -0.32195100 0.84945700

H 2.33500800 -1.43584300 2.28398400

H 2.74614100 -2.26230900 0.32290900

P -1.55640700 1.03044600 -1.08150400

P 1.12644100 2.18110200 0.36372300

H 0.71792300 2.93584300 1.50159000

H 2.54263900 2.30579300 0.47038000

H 0.86505700 3.11742200 -0.67687900

P -0.50600000 -1.95149600 -1.03736100

H -0.75861600 -1.86868900 -2.43508000

H 0.35121700 -3.09158900 -1.00725200

H -1.72265600 -2.52200000 -0.56552900

C 1.46677100 0.21594600 -1.89348000

H 2.36298400 0.83681500 -1.77565600

H 1.83164900 -0.74890400 -2.26600400

H 0.87303500 0.66586100 -2.69518600

Ir 0.26764400 0.01613700 -0.02015100

**1q-TS1**

E = -330.2616772 a.u.

C 1.78369700 -0.29619000 -2.06348200

C 0.43926000 -0.31268300 -1.81933100

H 2.12317100 -0.50474000 -3.07744900

H -0.18909100 -0.60063900 -2.66663200

C 2.83059500 0.38698200 0.19707800

C 2.84287100 -0.03977500 -1.12111300

H 3.80376500 0.55437800 0.65779800

H 3.84210100 -0.16339200 -1.54166900

P -2.34378000 -1.27429800 -0.32077500

H -3.58170100 -0.56903300 -0.41923000

H -2.47002100 -2.07253500 -1.50108400

H -2.75282600 -2.25884800 0.63180200

P 1.42843700 1.28460200 0.86540600

P -1.36591400 2.05314200 -0.50247500

H -1.01891700 2.65850600 -1.74432500

H -2.78959900 2.08561700 -0.60069700

H -1.15058800 3.15117000 0.38202300

P 0.83845100 -1.97681700 0.66985800

H 1.67459800 -1.91682900 1.81909900

H 0.01735200 -3.08676700 1.03586300

H 1.69628000 -2.60318000 -0.27649000

C -0.19063500 0.42863200 2.38241600

H -0.57988500 1.40480800 2.67321500

H -0.92852600 -0.34451600 2.62576400

H 0.68166000 0.21226800 3.01128400

Ir -0.29831700 0.02276000 0.03710500

**1q-IN1**

E = -330.3103443 a.u.

C -1.13125100 0.27922400 2.40575700

C 0.05073400 0.17072900 1.76851800

H -1.04865100 0.35119300 3.49151700

H 0.96637400 0.23573600 2.36182600

C -2.86553000 0.34115000 0.59269700

C -2.48607500 0.27951900 1.88986600

H -3.93253200 0.32983400 0.37173000

H -3.27003300 0.22773300 2.64278100

P 2.85511000 -0.57040200 0.10565600

H 3.86807600 0.43766600 0.12547700

H 3.20756700 -1.23303700 1.32173900

H 3.49033700 -1.46830000 -0.80504100

P -1.80355600 0.62506500 -0.85197800

P 0.75812000 2.34647600 -0.30569400

H -0.24745300 3.10700500 0.35388800

H 1.94338200 2.95691500 0.21286100

H 0.72374600 2.93071100 -1.60457600

P -0.27646800 -2.24280500 0.07770700

H -0.42796600 -3.09344500 -1.05768900

H 0.50598500 -3.09503100 0.91138600

H -1.55199400 -2.37447500 0.69349300

C -2.46890600 -0.67689700 -2.03046600

H -3.43038300 -0.30737100 -2.40161100

H -1.80432000 -0.76551000 -2.89374000

H -2.63628000 -1.66097000 -1.58617000

Ir 0.44223700 -0.01709100 -0.19846100

**2q**

E = -330.3313746 a.u.

C 2.39880900 1.54255600 -0.50955300

C 1.05302300 1.58699700 -0.72711300

H 3.01227200 2.35497200 -0.89782800

H 0.69267100 2.52759800 -1.16071200

C 2.54890400 -0.44196700 1.00344200

C 3.10789800 0.51408800 0.20397700

H 3.20143300 -1.20414100 1.42775400

H 4.19108700 0.50771600 0.09071000

P 0.85527200 -0.40954800 1.61549300

P -2.25907300 -1.32059400 -0.08473400

H -3.37967900 -1.15612300 -0.95520400

H -2.07850200 -2.72686800 -0.26079800

H -2.97668100 -1.37234400 1.15040400

P 0.80406900 -1.26747700 -1.70981600

H 1.02873300 -2.61004300 -1.28238700

H 0.22419900 -1.53271800 -2.98726800

H 2.11480300 -0.87701000 -2.09750300

P -1.50104700 1.93906200 0.91062500

H -2.41914600 2.80566900 0.24185200

H -2.30479700 1.56318400 2.02659700

H -0.65574200 2.90278500 1.52924300

C 0.62036800 -2.21864500 2.02012800

H 0.84129100 -2.89804100 1.19420000

H 1.29425600 -2.45720100 2.85010800

H -0.39949700 -2.38690600 2.37427800

Ir -0.37985700 0.21480100 -0.33977000
